# Influence of the Intervertebral Disc Microenvironment on Matrix Synthesis and Metabolism in Goat Nucleus Pulposus Cells

**DOI:** 10.1002/jsp2.70160

**Published:** 2026-01-14

**Authors:** Niamh Wilson, Tara Ní Néill, Jake McDonnell, Emily McDonnell, Pieter A. J. Brama, Conor T. Buckley

**Affiliations:** ^1^ Trinity Centre for Biomedical Engineering, Trinity Biomedical Sciences Institute, Trinity College Dublin The University of Dublin Dublin Ireland; ^2^ Discipline of Mechanical, Manufacturing and Biomedical Engineering, School of Engineering, Trinity College Dublin The University of Dublin Dublin Ireland; ^3^ Advanced Materials and Bioengineering Research (AMBER) Centre, Royal College of Surgeons in Ireland & Trinity College Dublin The University of Dublin Dublin Ireland; ^4^ National Spinal Injuries Unit Mater Misericordiae University Hospital Dublin Ireland; ^5^ School of Veterinary Medicine University College Dublin Dublin Ireland; ^6^ Tissue Engineering Research Group, Department of Anatomy and Regenerative Medicine RCSI Dublin Ireland

**Keywords:** glucose, in silico modeling, intervertebral disc, metabolism, microenvironment, nucleus pulposus, osmolarity, oxygen, pH, synthesis rates

## Abstract

**Background:**

The intervertebral disc (IVD) microenvironment plays a crucial role in cellular function and viability. Although the precise cause of IVD degeneration remains unclear, it is associated with progressive disruption of nutrient, metabolite, and pH homeostasis. Despite growing interest in regenerative therapies, the complex IVD microenvironment is often overlooked in preclinical development. This study investigates the effects of clinically relevant combinations of oxygen, glucose, pH, and osmolarity on the metabolic activity and matrix synthesis of goat nucleus pulposus (NP) cells.

**Materials and Methods:**

Goat NP cells were embedded in 3D alginate beads and exposed to 24 distinct microenvironments across four factors in combination: oxygen (2% and 5%), glucose (0.5 and 1.0 mM), pH (6.5, 6.8, and 7.1), and osmolarity (350 and 500 mOsm). Alginate beads were primed for 10 days before subjection to altered microenvironmental conditions for a further 14 days. Cell viability, DNA content, glycosaminoglycan (GAG), and collagen synthesis, as well as oxygen consumption and lactate production rates, were quantified. Experimental data informed in silico modeling of a goat IVD, profiling nutrient and metabolite gradients and GAG accumulation to determine the effects of microenvironmental changes at the whole‐organ level.

**Results:**

pH was the most influential factor, significantly reducing cell viability, DNA content, and GAG production under degenerated conditions at pH 6.5. Collagen production remained unchanged. Oxygen and glucose significantly affected metabolic rates. Combined analysis revealed the interdependent nature of these factors, better reflecting in vivo interactions. In silico modeling demonstrated that microenvironment‐driven changes directly altered disc‐wide nutrient profiles and long‐term GAG accumulation.

**Conclusion:**

These findings highlight the critical role of pH in regulating NP cell function and show that interactions between microenvironmental factors impact cell behavior more than isolated effects. Incorporating physiologically relevant microenvironments may improve regenerative therapy development and enhance translation from preclinical models to clinical application.

## Graphical abstract

Goat nucleus pulposus cells cultured under clinically relevant oxygen, glucose, pH, and osmolarity revealed pH to have the most influential impact on viability and glycosaminoglycan synthesis. Interactions between factors showed that disc cell behavior is shaped by the microenvironment, highlighting its importance in regenerative therapy design for the intervertebral disc.

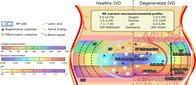

## 1 Introduction

Although the precise cause of intervertebral disc (IVD) degeneration remains unclear, the intradiscal microenvironment has long been established to play a crucial role in cell function and viability [1, 2]. Owing to its avascular nature, the IVD is highly dependent on the diffusion of nutrients and metabolites through the cartilage endplate (CEP) [3, 4]. The CEP serves as an essential pathway through which vital substances such as oxygen, glucose, and other nutrients enter the disc, thereby supporting the metabolic activity of the IVD cells [5]. This pathway also facilitates the removal of metabolites; if this route is impaired, lactic acid can accumulate leading to a reduction in the pH of the IVD. Upon degeneration, calcification of the CEP can lead to inhibition of the diffusion pathways, altering this niche microenvironment. This perturbation in nutrient and metabolic exchange can further exacerbate disc degeneration, leading to diminished cell viability and further loss of function [6].

Recent advancements in regenerative therapies have focused on restoring the disc's native function by promoting matrix synthesis, reducing cell death, and preserving its load bearing capacity [7, 8, 9, 10, 11]. However, the development of these therapies often overlooks the importance of the complex and dynamic nutrient microenvironment within the IVD, which may hinder their success in clinical settings. A recent systematic review highlights this discrepancy in commonly used culture conditions. McDonnell et al. reviewed 55 studies on NP cell culture and found that the most commonly used glucose concentrations were 25 mM and 5.55 mM, with 25 mM used in 62.5% of studies. Reported oxygen concentrations included 21% (normoxia, 44.8% of studies), 5% (physoxia, 34.5% of studies), and 2% (hypoxia, 20.7% of studies). Basal culture media typically have an osmolarity of 310–340 mOsm with an initial pH of 7.0–7.4. When exposed to atmospheric CO_2_, dissolved CO_2_ diffuses out of the media, altering the sodium bicarbonate buffer equilibrium, causing the pH to rise.

The microenvironment of the IVD has been previously characterized across various states of degeneration for both human and goat NP tissue. These studies have measured or predicted key parameters including oxygen, glucose, osmolarity, and pH, all of which play a role in maintaining a balanced cellular environment. Nutrients and metabolites form gradients across the IVD due to their diffusional distance from the CEP, with these gradients becoming more distinct as degeneration progresses [12, 13, 14]. Notably, in silico or computational models have demonstrated that gradients of oxygen, glucose, and lactate are amplified in more degenerated cases, creating a less favorable microenvironment for cellular function [3, 13, 15].

**FIGURE 1:**
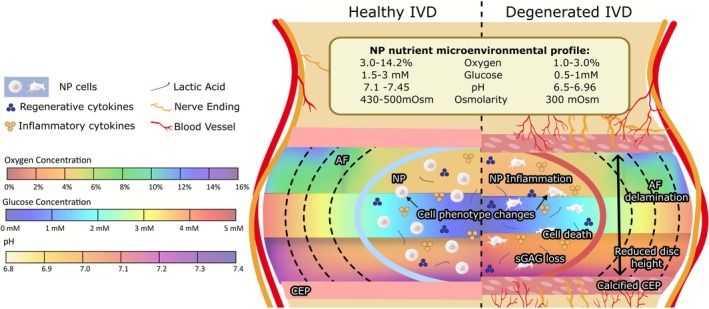
Microenvironment of the goat intervertebral disc illustrating oxygen, glucose, and lactate profiles across the disc in both healthy and degenerated states. All species are denoted to decrease upon degeneration with physical changes including CEP calcification and reduced disc height acting as contributing factors to this change in homeostasis; range of values of each species outlined above for healthy and degenerative cases.

The innermost part of the nucleus pulposus (NP) represents the longest diffusion pathway from the CEP resulting in the central region of the IVD being the most deprived of nutrients and concentrated in metabolites. Key studies have found oxygen levels in the human NP region to range from 2% to 10%, with a mean value of 6% (0.0185–0.0925 mM) [16]. Oxygen concentration in goat NP tissue has been shown to fall in a similar range using in silico modeling [17], with values ranging from 7.25 to 7.1 in healthy NP tissue. However, recent profiling from an in vivo degeneration model reported oxygen concentrations to range from 6.6% to 14.2%. This could be attributed to changes in endplate porosity or disc height that have not been captured in computational models [18]. Glucose concentration within human NP tissue has not been experimentally determined, with current values predicted through in silico modeling ranging from 1 to 5 mM [19, 20]. Similarly, glucose concentrations in goat NP tissue have also been profiled to fall within this range at 1–2 mM [17]. Owing to the lack of experimental profiling and reliance on in silico modeling across species, a conservative lower glucose level of 0.5 mM was included in this study as it has been previously shown to impact cellular function at this level [21]. Osmolarity in healthy human NP tissue ranges from 430 to 500 mOsm, decreasing to 300 mOsm in degenerated tissue [22]. To the best of our knowledge no studies have yet profiled the osmolarity in goat NP tissue; however, studies in other large animals such as bovine tissue have found the NP osmolarity to fall within a similar range to that of human NP [23]. pH ranges from 7.1 in healthy human NP tissue to 6.8 in mildly degenerated tissue, further decreasing to 6.5 in the case of moderate to severe degeneration [16]. Although pH in goat NP tissue has not explicitly been measured, a recent study reported lactate levels to fall between 0.465 and 5.379 mM corresponding to a pH of approximately 7.45–6.96 [18]. Similar to glucose concentrations, profiling of pH in severely degenerated caprine discs is limited and therefore reliance on human measurements was required to establish a lower boundary condition. For these purposes 6.5 was chosen as it was found to be a common lower value in grade IV human discs [24]. These changes in the microenvironment as degeneration progresses are indicative of a shift toward a more acidic, hypoxic, and nutrient‐poor environment, which could have significant implications for the cellular health and function of disc cells. The range of nutrient microenvironments established for a healthy and degenerated goat IVD are summarized in Figure 1.

Understanding the cellular response to this dynamic microenvironment is crucial for the development of precision based regenerative therapies in vitro. This area has been explored in relation to many factors within the disc across various species [21, 25, 26, 27]. To better investigate these microenvironmental effects in a controlled setting, caprine discs have emerged as a suitable animal model due to their comparable size, load‐bearing function, and cellular metabolism to human IVDs [28]. Previous work in our laboratory has demonstrated that the viability and metabolic rates of native caprine cells in vitro are comparable to those of human cells [17]. Using both in silico modeling and experimental measurements, we have also shown that the oxygen, glucose, and pH microenvironments fall within a similar range, indicating that the cells and ECM of goat IVDs provide a suitable alternative to mildly degenerated human sources [17].

Cell viability has been shown to be adversely affected by reduced glucose levels (less than 0.5 mM) sustained for more than 3 days [29], and pH values of less than 6.5 [30, 31, 32]. Oxygen plays a key role in cellular ATP production; however, due to their location in the most hypoxic region of the disc, NP cells have adapted to rely primarily on glycolysis for energy [21, 27], even in the presence of ambient oxygen conditions. This adaptation indicates that in cases of poor oxygen availability coupled with low glucose levels, cellular function may be compromised; although the combined impact of these factors remains to be fully elucidated.

To date, most studies examining the relationship between cellular synthesis and metabolic rates in response to microenvironmental factors have investigated these influences independently of one another [27, 33, 34, 35]. Within the disc, cellular responses depend on the interplay of these factors which vary widely based on individual anatomy and the degree of degeneration. The complex nature of this microenvironment necessitates a better integrated approach to studying how multiple factors interact and how they collectively influence cellular behavior. Therefore, the objective of this study is to elucidate the combined effects of oxygen, glucose, pH, and osmolarity, within a clinically relevant range, on the matrix synthesis and metabolic rates of goat NP cells. By examining these factors in combination, this work aims to enhance our understanding of the potential responses of regenerative therapies in vivo.

## 2 Materials and Methods

### 2.1 Experimental Design

**FIGURE 2:**
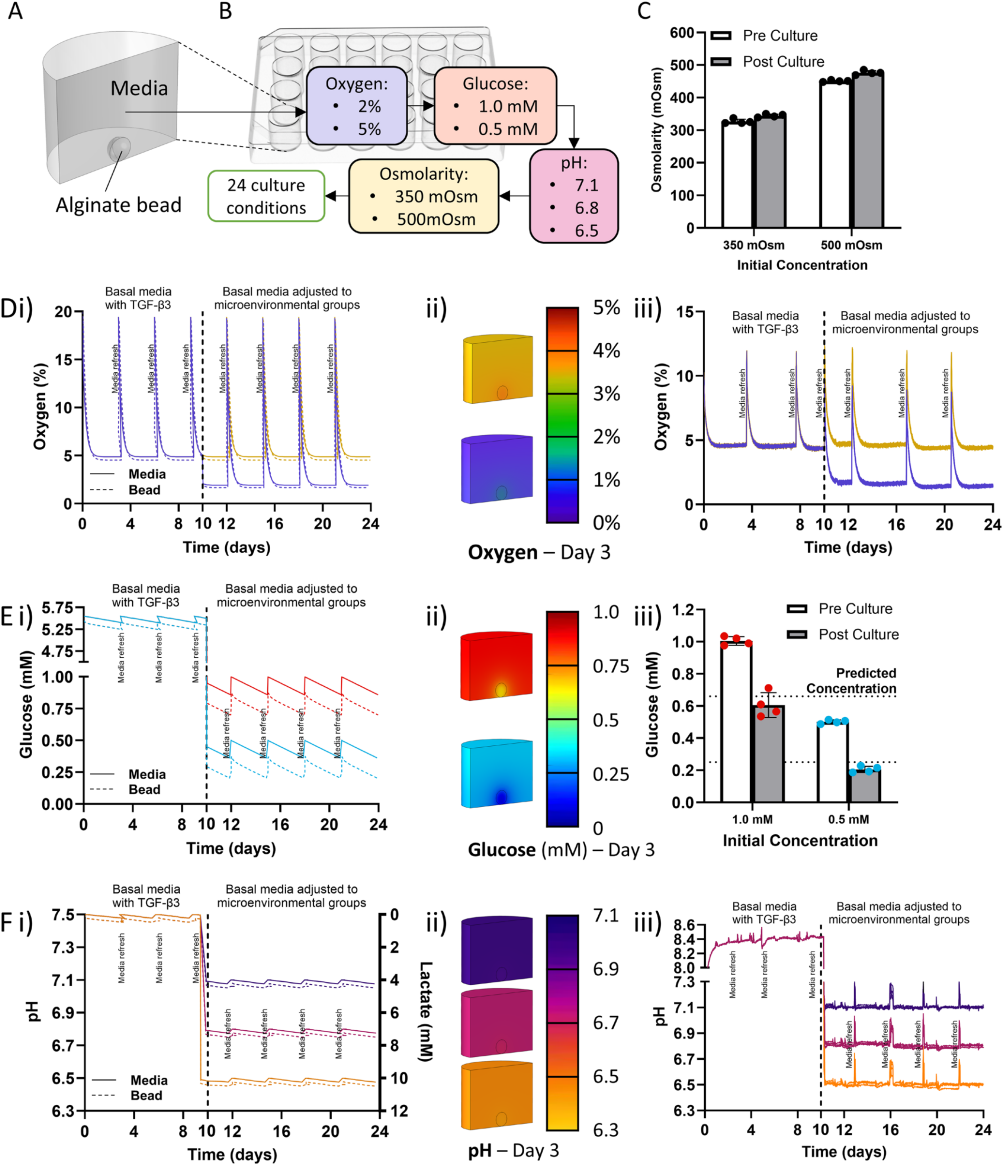
Experimental set up and validation of media conditions. (A) Culture and in silico model set up containing a single alginate bead per well of a 24 well plate. (B) Flow chart detailing experimental groups for Oxygen (2% and 5%), Glucose (0.5 and 1.0 mM), pH (6.5, 6.8, and 7.1), and Osmolarity (350 and 500 mOsm) which when taken in combination results in 24 experimental groups. (C) Experimental validation of Osmolarity pre and post 3 days in culture, after which time media change occurs. (D–F) Oxygen, Glucose and pH experimental conditions respectively. (i) predicted concentrations from in silico modeling, (ii) contour plots visualizing in silico modeling concentrations post 3 days in culture. (iii) experimental validation of culture conditions.

An alginate bead culture model was used to assess the impact of clinically relevant oxygen, pH, glucose, and osmolarity levels on the matrix synthesis and metabolic rates of caprine NP cells [36]. Post expansion to P2, primary caprine NP cells were seeded in alginate beads at a cell density of 2.5 × 10^6^. Alginate beads were primed for 10 days with TGF‐*β*3 to provide a protective extracellular matrix (ECM) niche [24, 30] around the cells to more closely mimic the in vivo scenario. During this period, cultures were maintained at 5% oxygen in low‐glucose DMEM (5.55 mM glucose). Media pH and osmolarity were not experimentally altered and therefore reflected standard DMEM conditions (approximately 7.0–7.4 pH and 310–340 mOsm), which gradually increased to pH 8.2–8.4 over time due to buffering effects, as shown in Figure 2. Beads were subsequently subjected to simulated healthy and degenerate microenvironments for 14 days by altering oxygen, glucose, pH, and osmolarity levels in the media within a clinically relevant range as defined from the literature. Oxygen concentrations of 2% and 5% were chosen based on measurements and microenvironmental profiling previously conducted by McDonnell et al. [17], as it has been previously shown that low oxygen microenvironments can impact cellular behavior [37, 38]. Glucose concentrations were also determined from in silico modeling, with the lower group defined at 0.5 mM as a result of the findings from Horner and Urban who reported compromised cell viability after 3 days at this level [26]. Although osmolarity has not been directly profiled in goat NP tissue, bovine NP osmolarity falls within that of the human range and has a similar tissue consistency to that of goat NP. Therefore, healthy and degenerated human measurements were used to define two osmolarity microenvironments; 500 mOsm (healthy) and 350 mOsm (degenerated) [22]. Finally, three pH levels were defined based on human measurements by Bartels et al. and from in silico modeling of goat nutrient environments [16, 17]. The most acidic pH group (6.5) was selected to reflect the lower limit of these predicted gradients under degenerative conditions. Combining each of these factors resulted in 24 distinct microenvironments as outlined in Table 1. Beads were cultured individually in 24 well plates, with each well containing one bead and 2 mL of media per well. Complete media changes were performed every 3–4 days to maintain the microenvironmental conditions and minimize gradient formation within the 3D alginate constructs, as determined through in silico modeling as previously outlined [13]. Media was refreshed at 20% oxygen, exposing the beads to a brief oxygen fluctuation as indicated in the predicted modeling and experimental measurements in Figure 2D,i,iii. Microenvironmental stability was further validated experimentally. The 3–4‐day media‐change interval was established using in silico modeling and confirmed by measuring media composition before and after culture, which verified that oxygen, glucose, pH, and osmolarity remained within expected ranges. In silico diffusion analyses also predicted minimal spatial gradients within and around the alginate beads, indicating that the desired local microenvironmental conditions were effectively maintained throughout culture. These results are illustrated in Figure 2.

**TABLE 1:** Experimental culture conditions defined based on literature‐derived microenvironmental profiles of oxygen, pH, glucose, and osmolarity.

Oxygen (%)	pH	Glucose (mM)	Osmolarity (mOsm)	Culture group
2%	6.5	0.5	350	1
			500	2
		1.0	350	3
			500	4
	6.8	0.5	350	5
			500	6
		1.0	350	7
			500	8
	7.1	0.5	350	9
			500	10
		1.0	350	11
			500	12
5%	6.5	0.5	350	13
			500	14
		1.0	350	15
			500	16
	6.8	0.5	350	17
			500	18
		1.0	350	19
			500	20
	7.1	0.5	350	21
			500	22
		1.0	350	23
			500	24

### 2.2 Cell Isolation and Monolayer Expansion

NP tissue was isolated from the lumbar discs of mature (4–5 years) female Saanen goat spines. Following best practice from the 3 R's to reduce animal use, spines were acquired from discarded materials from goats undergoing procedures from another animal study authorized by the University College Dublin Animal Research Ethics Committee (AREC‐P‐18‐17) and the Irish Health Products Regulatory Authority (HPRA AE18982/P142). Six lumbar discs were isolated and pooled per goat to form one donor, with three goat spines isolated in total. All discs were visually inspected for signs of degeneration prior to cell isolation; only tissue that appeared visibly hydrated and white in color was included. NP tissue was isolated from the spine in a sterile manner and incubated overnight to ensure sterility at 37°C in low glucose Dulbecco's Modified Eagle Medium (LG DMEM, Sigma‐Aldrich, Merck Life Science Ltd., U.K.) containing 2% penicillin/streptomycin (Pen/Strep, Invitrogen, Bio‐Sciences Ltd., Ireland), and 10% Fetal Bovine Serum (FBS, Invitrogen, Bio‐Sciences Ltd., Ireland), 100 μg/mL kanamycin sulphate (Bio Sciences Ltd., Ireland) and 0.25 μg/mL Amphotericin‐B (Sigma‐Aldrich, Merck Life Science Ltd., U.K).

Tissue was enzymatically digested in LG DMEM supplemented with 2% Pen/Strep, 300 U/mL Collagenase Type II (Gibco Invitrogen, Dublin, Ireland) and 100 U/mL pronase (Sigma‐Aldrich, U.K) for 4–6 h under constant rotation at 37°C. Tissue dissociation was conducted hourly using the gentleMACS tissue dissociator (Miltenyi Biotech, United Kingdom) until the tissue was visibly digested, and single cells were visible under a microscope. Cell suspension was passed through a 70 μm cell strainer to remove any remaining debris. Cells were seeded at 5 × 10^3^ cells/mm^2^ in T175 flasks and expanded to passage 2 in LG DMEM supplemented with 2% Pen/Strep and 10% FBS in a humidified atmosphere at 37°C, 5% CO_2_ and 5% O_2_, with media changes performed every 3 days. The inclusion of 10% FBS during the expansion phase is consistent with current consensus recommendations [39], which acknowledge that limited serum supplementation is often required to obtain adequate cell numbers before experimental culture. Thereafter, cells were maintained in a basal medium without FBS for the remainder of the study.

### 2.3 Media Preparation and Stabilization

Zero glucose DMEM supplemented with 100 U/mL penicillin, 100 μg/mL streptomycin, 1 × ITS (all GIBCO, Invitrogen, Dublin, Ireland), 0.25 μg/mL Amphotericin B, 40 μg/mL L‐proline, 1.5 mg/mL BSA, 4.7 μg/mL linoleic acid, 50 μg/mL L‐ascorbic acid‐2‐phosphate, 100 nM dexamethasone (all Sigma‐Aldrich, Ireland) was used as a basal media for all conditions. Glucose concentrations were adjusted with the addition of D + glucose (Sigma‐Aldrich, Ireland) to 0.5 mM (90.08 μg/mL) and 1 mM (180.16 μg/mL). Osmolarity of the base media after supplementation was recorded at 326.5 mOsm, N‐Methyl‐d‐glucamine hydrochloride (Fluorochem, Ireland) was added to increase the osmolarity of the media to 350 and 500 mOsm (0.5 mM N‐Methyl‐d‐glucamine hydrochloride/mOsm). This compound is considered inert and does not biochemically interfere with cellular function [40]. Media was adjusted to three different pH values (6.5, 6.8, and 7.1) as previously described [30] through the addition of 3 mM HCL (0.5 μL/0.1 decrease in pH), and incubation in upright T75 flasks overnight in a humidified atmosphere at 37°C, 5% CO_2_, and 5% O_2_. pH stabilization was monitored in real time via a non‐invasive fluorescence‐based SensorDish Reader (SDR) (PreSens Germany). Measurements obtained using the SDR plate reader during the TGF‐*β*3 priming phase (days 0–10) indicated that, due to the buffering capacity of the medium as a result of sodium bicarbonate and carbon dioxide levels, the pH increased to between 8.2 and 8.4 (Figure 2F,iii). Glucose and osmolarity were verified after 3 days by biochemical assay (Sentinel Diagnostics, Italy) and vapor pressure osmometer (Elitech, UK), respectively (Figure 2).

### 2.4 Alginate Encapsulation and Culture

Expanded NP cells were encapsulated in 1.5% alginate (Pronova UP LVG, FMC NovaMatrix, Sandvika, Norway) at a seeding density of 2.5 × 10^6^ cells/ml and ionically crosslinked using a needle dropping method (20G) with 100 mM calcium chloride (SLS Scientific Laboratory Supplies (Ireland) Ltd) for 20 min at 37°C to form spherical beads. A seeding density of 2.5 × 10^6^ cells/mL was selected as it falls within reported NP cell density ranges for both human and goat discs and is consistent with established alginate NP culture protocols [41]. Beads were primed for 10 days in basal media with 5 mM glucose (D + glucose) and 10 ng/mL TGF‐*β*3 (PeproTech, UK) to provide a protective matrix niche [24, 30]. Beads were cultured for a further 14 days in the 24 microenvironmental combinations defined in the experimental design (Table 1). All cultures were conducted in a humidified atmosphere at 37°C, 5% CO_2_ with media changes performed every 3–4 days to maintain experimental conditions within the range of the IVD microenvironmental groups. Media was collected after each exchange for biochemical analysis. Three independent experiments were performed, each using cells from a different biological donor (total *n* = 3). Six beads were used per donor: one for viability, three for biochemical assays (averaged as technical replicates), and two for histology.

### 2.5 Assessment of Cell Viability

Cell viability was assessed via a live/dead assay. Media was aspirated and beads were washed with phenol free DMEM before incubation for 1 h at 37°C in a phenol free low glucose‐DMEM solution containing 2 μM calcein (Sigma‐Aldrich, Merck Life Science Ltd., U.K) and 4 μM ethidium homodimer‐1 (EthD‐1) (SLS Scientific Laboratory Supplies (Ireland) Ltd). After incubation the staining solution was aspirated, and beads were washed three times with phenol free media. Beads were imaged on a Leica SP8 scanning confocal microscope (485 and 530 nm excitation and 530 nm and 645 nm emission for calcein and EthD‐1 respectively). Images were analyzed using ImageJ software (version 1.53e) to generate maximum projection z‐stack images, enabling qualitative assessment of cellular viability within the beads.

### 2.6 Quantitative Biochemical Analysis

Media was collected at every media change and lyophilized overnight at the 24‐day timepoint in a Labconco Triad freeze drier (Labconco Corporation, USA) using a standard procedure (500 mTorr, −10°C, 16‐18 h), before resuspension in 1 mL deionized water. Alginate beads were digested in papain (125 μg/mL) (SLS Scientific Laboratory Supplies (Ireland) Ltd) with 5 mmol/L L‐cysteine HCL (Sigma‐Aldrich, Merck Life Science Ltd.), papain buffered extract (PBE) (50 mM sodium phosphate dibasic anhydrous, 50 mM sodium phosphate monobasic anhydrous, 0.05 mol/L EDTA (all Sigma‐Aldrich, Merck Life Science Ltd.)) at 60°C for 18 h under constant rotation. Following this, samples were further incubated for 1 h with 1 M sodium citrate under constant agitation to break down ionic crosslinks.

DNA was quantified using the Quant‐iT Pico Green dsDNA kit (Thermo Fisher Scientific, Ireland). Sulphated glycosaminoglycan (sGAG) content was quantified through dimethylene blue dye‐binding assay, using a chondroitin sulfate standard (Shark) (all DMMB Blyscan, Biocolour Ltd., UK) [30, 42, 43]. This quantification method binds dye to GAGs, producing a measurable color after dissociation proportional to the GAG concentration. A 100 μL aliquot from the 200 μL digest was used to obtain readings within the standard curve. Beads cultured at pH 6.5 were pooled to ensure sufficient GAG content for quantification. Collagen was quantified using a hydroxyproline assay, which quantifies a collagen‐specific amino acid, hydroxyproline, which can then be converted to collagen content by applying a hydroxyproline to collagen conversion ratio of 1:7.69 [30, 44, 45]. A 10 μL aliquot from the 200 μL digest was used to obtain a reading within the standard curve, with beads cultured at pH 6.5 pooled to ensure sufficient hydroxyproline content for quantification.

### 2.7 Histological Staining

Samples were fixed in 4% paraformaldehyde (PFA) overnight and dehydrated in increasing ethanol solutions and wax embedded in paraffin wax. Beads were sectioned at 10 μm on a microtome (Leica Microsystems, Germany) and stained for tissue structure (hematoxylin and eosin) [17], sulfated ECM components including sGAG (aldehyde fuchsin), and collagen (picrosirius red) [30, 46]. Slides were imaged at 20× magnification on a Leica Aperio AT2 slide scanner.

### 2.8 Metabolic Consumption Rates

Oxygen consumption rates (OCR) and lactate production rates (LPR) were quantified through the Seahorse XFe24 analyzer and islet capture microplates (Agilent Technologies, Ireland). The seahorse instrument was housed in a hypoxic chamber enabling quantification of metabolic rates at the corresponding oxygen concentrations of the culture groups (2% and 5%). Three alginate beads were included in each well to ensure sufficient reduction in oxygen for OCR measurements and increase in pH for Extracellular Acidification Rates (ECAR) measurements (correlated to LPR). Unlike the long‐term culture setup (one bead per well), this assay was short in duration; therefore, effects of gradient formation on metabolic rates were minimized. Seahorse cartridge plates were incubated in DI water in a non‐CO_2_ incubator at 37°C for a minimum of 8 h before use to remove any residual CO_2_ that could affect measurements. DI water was then exchanged for XF calibrant 30 min before running the assay. Each well was filled with three alginate beads capped with a small mesh insert to prevent floating. Each well was then filled with 1 mL of unbuffered Seahorse XF DMEM supplemented with 1 mM sodium pyruvate, 2 mM L‐glutamine and glucose to 0.5 or 1.0 mM (all Agilent Technologies Ireland) depending on the conditions of the culture group. Media pH was adjusted to 6.5, 6.8, or 7.1 corresponding to each culture group. Blank wells were prepared for each media combination to remove background OCR and ECAR in each case. Prior to measuring, each plate was placed inside the hypoxia chamber and incubated along with the sensor cartridge and Seahorse analyzer to the required oxygen concentration. Measurements were performed 3 times at 20‐min intervals under basal conditions—no injection of inhibitor or stimulator treatments. Following the seahorse assay, samples were removed from the plate and digested in papain enzyme solution and quantified for DNA as described above for normalization purposes. DNA content was converted to cell number using a defined standard curve. Raw measurements for oxygen (mmHg) and pH were extracted from wave software and data analysis was performed in Excel. pH measurements were converted to lactate employing a previously defined linear relationship [47, 48]. All measurements were normalized by cell number per well.

### 2.9 In Silico Modeling

In silico modeling was conducted using COMSOL Multiphysics 6.2 (COMSOL Inc., Burlington, USA), to predict nutrient and metabolite gradients across oxygen, glucose, and pH. Models were based on previous work reported by McDonnell et al. [49] using the transport of dilute species physics derived from Fick's Law, applied across all domains and species. Since pH exhibited the strongest influence on cellular behavior, with OCR significantly affected by pH under 5% oxygen conditions, subsequent analyses focused on two representative microenvironments at 5% oxygen: (1) the most degenerated condition (pH 6.5, 0.5 mM glucose, 350 mOsm osmolarity) and (2) the healthiest condition (pH 7.1, 1.0 mM glucose, 500 mOsm osmolarity). An idealized geometry was created using measurements from goat lumbar discs (L3‐4) as previously described [17].

Active cell densities employed in the model were defined based on the total cell density of NP tissue as previously described [41], scaled by the viability observed in this study (40% and 80% for degenerated and healthy groups respectively). The specific cell densities assigned to each region and condition are summarized in Table 2.

**TABLE 2:** Cell density values used in the in silico models for degenerated and healthy goat intervertebral disc tissue (NP and annulus fibrosus (AF) regions).

	Degenerated (cells/mm^3^)		Healthy (cells/mm^3^)	
	NP	AF	NP	AF
Total cell density	6133	19732	6133	19732
Viability	40%		80%	
Active cell density	2453.2	7892.8	4906.4	15785.6

Metabolic rates were derived from experimental measurements of OCR and LPR conducted in this study. These values were used to simulate how NP cell metabolism, modulated by different microenvironmental conditions, contributes to shaping the surrounding nutrient and metabolite gradients within the disc. Glucose consumption rate was inferred from the LPR using an established coupled reaction diffusion equation based on the conversion of glucose to lactate under anaerobic glycolysis. This conversion employed a 2:1 ratio where GCR was calculated as half the LPR.

Boundary conditions for each species were determined iteratively by adjusting the outer AF and CEP boundary, initially using concentrations as reported by McDonnell et al. [17] and adjusting until the models reflected the cell culture groups corresponding to the degenerated and healthy states. The final boundary values used for each simulation scenario are summarized in Table 3.

**TABLE 3:** Boundary conditions used for in silico models, established through iterative adjustment to achieve target NP concentrations for healthy and degenerated states.

	Degenerated		Healthy	
	AF	CEP	AF	CEP
Oxygen (%)	15	12	15	12
Glucose (mM)	1.4	0.8	4.0	2.3
Lactate (mM)	6.3	10.5	2.5	1.0

	NP	NP
OCR (nmol/million cells/h)	51.86	19.28
LPR (nmol/million cells/h)	61.47	133.63

*Note:* Metabolic rates experimentally determined in this study.

Finally, diffusion coefficients for oxygen, glucose, and lactate were established from previous studies and employed in this study for healthy and degenerated tissue as outlined in Table 4 [13].

**TABLE 4:** Diffusion coefficients for oxygen, glucose and lactate in the NP, CEP and AF tissue for both degenerated and healthy states.

	Common to both models			Degenerated	Healthy
		AF			
	NP	Radial	Axial	CEP	CEP
Oxygen (mm^2^/h)	3.08	3.08	2.2	1.74	3.53
Glucose (mm^2^/h)	0.45	0.37	0.45	0.06	0.12
Lactate (mm^2^/h)	0.61	0.61	0.5	0.1	0.21

A transient regeneration model of GAG within the NP was adapted from previous studies based on the conservation of mass for GAG theory [17]. In this study the GAG synthesis rates were experimentally determined from total GAG produced over a 24‐day period in both groups and are defined in Table 5. Initial GAG content as well as GAG degradation rates were established from previous studies and factored by viability as observed in this study, outlined in Table 5 [17].

**TABLE 5:** GAG parameters used to inform transient GAG synthesis model.

	Degenerated	Healthy
GAG synthesis rate (pg/cell/day)	25.9	25.4
GAG degradation rate (/year)	8.6%	8.6%

	NP	AF	NP	AF
Initial GAG (g/mm^3^)	94.8 × 10^−6^	26.9 × 10^−6^	94.8 × 10^−6^	26.9 × 10^−6^
Viability	40%		80%	
Scaled initial GAG (g/mm^3^)	37.9 × 10^−6^	10.8 × 10^−6^	75.9 × 10^−6^	21.5 × 10^−6^

### 2.10 Statistical Analysis

Statistical analysis was performed using R‐Studio (version 2024.12.0) and data graphed using GraphPad Prism (version 10). Data was tested for normality using a Shapiro–Wilk test. A four‐way ANOVA was used to determine the variance between groups, with Tukey's post hoc tests used to compare between all groups and account for adjusted *p*‐values. A Type III Wald chi‐squared analysis was used to compare the influence of all factors. Statistical significance was accepted as *p* ≤ 0.05. Each group contained three technical replicates across three individual donors (biological replicates), with each donor cultured in its own individual experiment. Results are displayed as mean ± standard deviation, with significance denoted by symbols above each bar. Specific details on significance levels can be found in Tables S1–S5.

## 3 Results

### 3.1 Interpretation of Graphs

**FIGURE 3:**
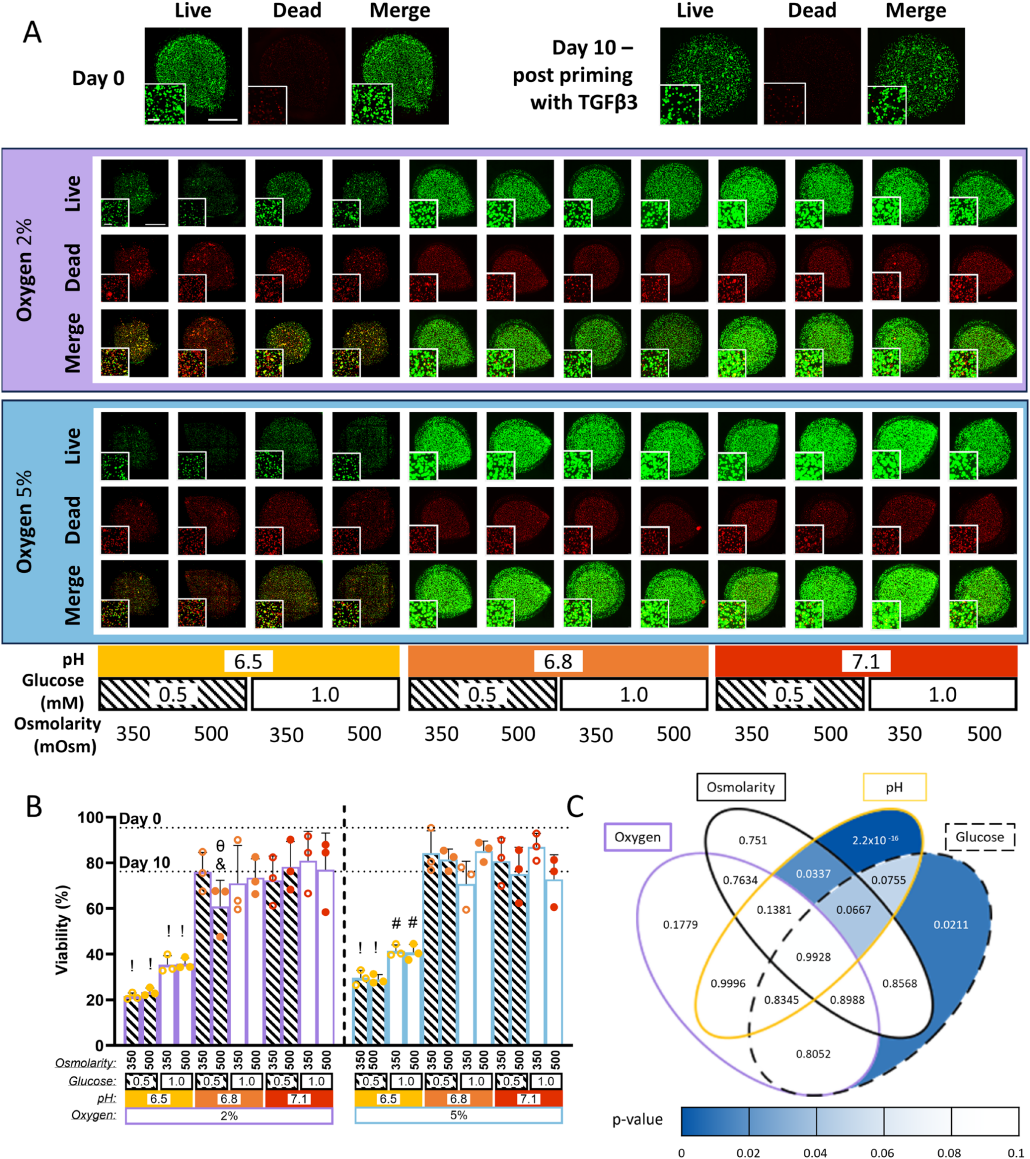
Viability of goat NP cells encapsulated in alginate beads subjected to various microenvironmental conditions. (A) Representative images showing cell viability at the initial timepoint (Day 0) and post 10 days priming with TGF‐*β*3 (Day 10). Below: Viability following exposure to 24 different culture conditions, comprising combinations of oxygen (2% or 5%), pH (6.5, 6.8, or 7.1), glucose (0.5 or 1.0 mM), and osmolarity (350 or 500 mOsm). Scalebar: 1 mm. (B) Bar chart showing cell viability after 10 days of TGF‐*β*3 priming followed by 14 days of culture under different microenvironmental conditions. “!” indicates significant reduction in viability compared to all pH 6.8 and 7.1 groups. “#” indicates significant reduction compared to all pH 6.8 and 7.1 groups excluding the 2% oxygen, pH 6.8, glucose 0.5 mM, and 500 mOsm group. (C) Venn diagram displaying *p*‐values from a four‐way ANOVA (*χ* ^2^ test). Heat mapping highlights significant effects and trends. pH, glucose, and the interaction between pH and osmolarity significantly affected cell viability (*p* = 2.2 × 10^−16^, 0.0211, and 0.0337, respectively). Full statistical details are provided in Table S1.

**FIGURE 4:**
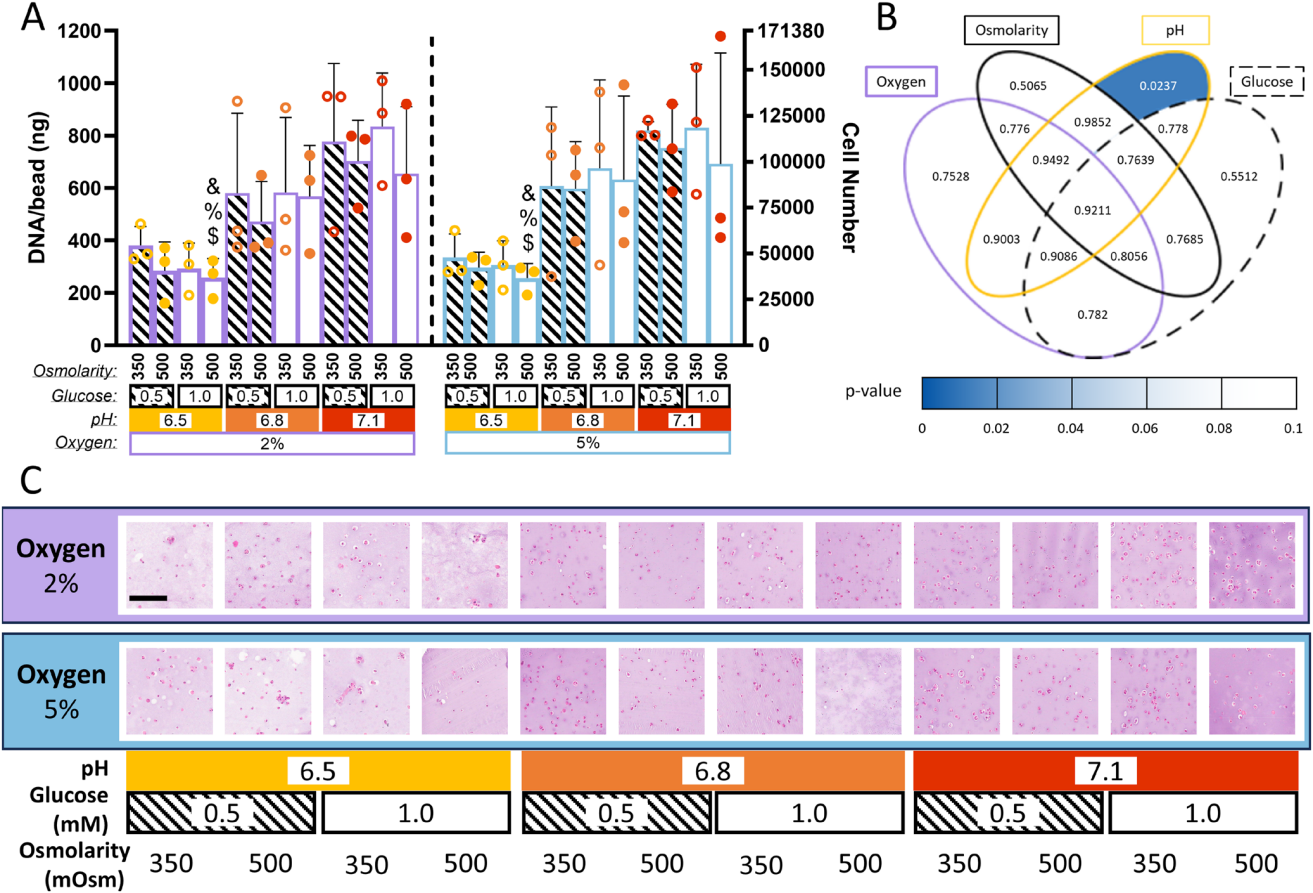
DNA content of goat NP cells encapsulated in alginate beads subjected to various microenvironmental conditions. (A) Bar chart showing DNA after 10 days of TGF‐*β*3 priming followed by 14 days of culture under different microenvironmental conditions (combinations of oxygen [2% or 5%], pH [6.5, 6.8, or 7.1], glucose [0.5 or 1.0 mM], and osmolarity [350 or 500 mOsm]). Significant decreases in DNA were noted in the pH 6.5 group at 1.0 mM glucose, 500 mOsm osmolarity at 2% and 5% oxygen when compared to three groups: 5% oxygen 7.1 pH, 0.5 mM glucose 350 mOsm osmolarity (*p* = 0.0485 and 0.0468 respectively, denoted by: &), 2% oxygen, 7.1 pH, 1.0 mM glucose 350 mOsm osmolarity (*p* = 0.0371 and 0.0358 respectively, denoted by: %) and 5% oxygen, 7.1 pH, 1.0 mM glucose and 350 mOsm osmolarity (*p* = 0.041 and 0.0396 respectively, denoted by: $) (B) Venn diagram displaying *p*‐values from a four‐way ANOVA (*χ* ^2^ test). Heat mapping highlights significant effects. pH significantly affected cell proliferation (*p* = 0.0237). Full statistical details are provided in Table S2. (C) H &E staining of alginate beads at day 24, across all culture conditions, lighter staining observed in the pH 6.5 group indicates lower matrix deposition. Scalebar: 200 μm.

**FIGURE 5:**
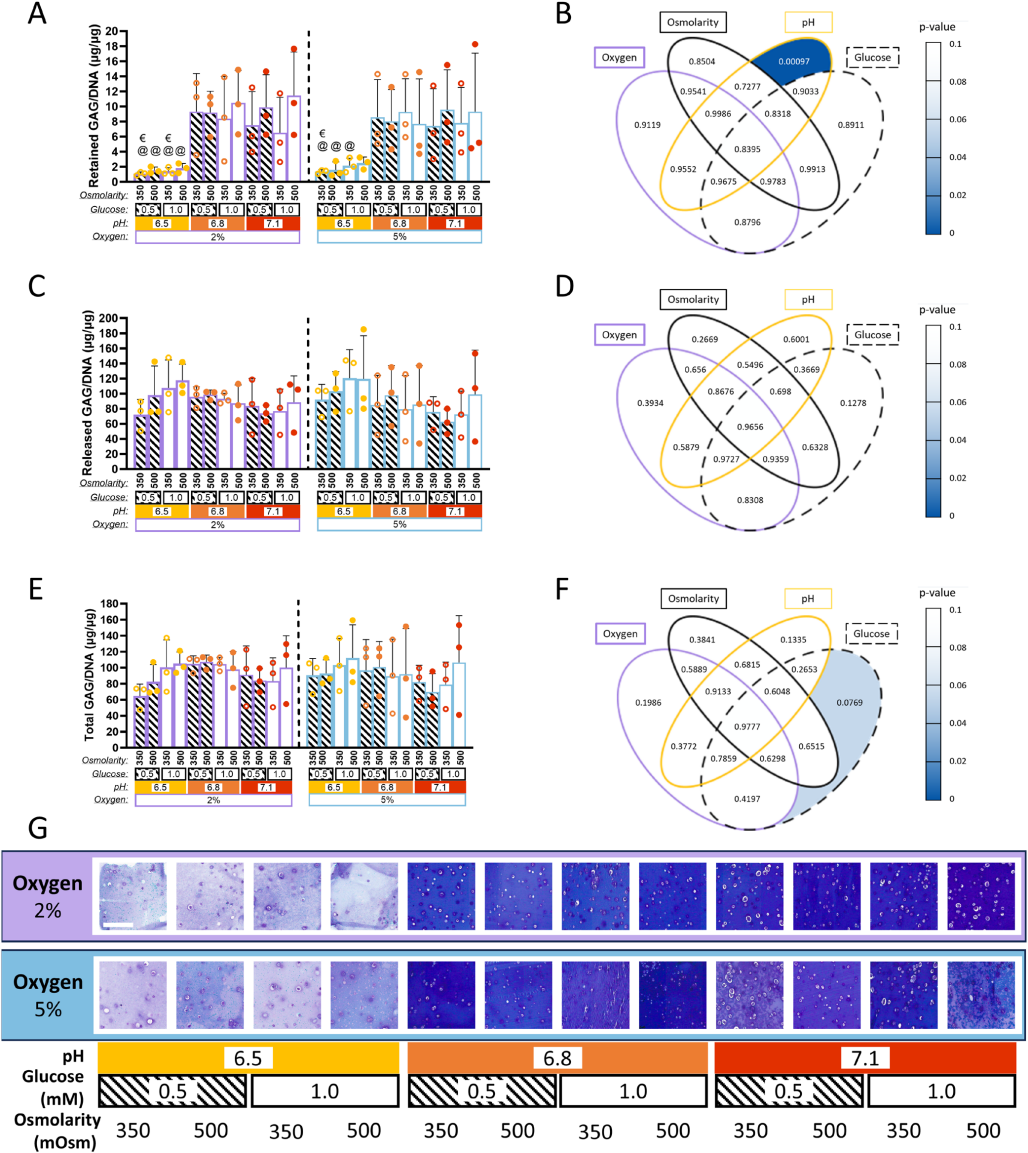
GAG production of goat NP cells encapsulated in alginate beads subjected to varied microenvironmental conditions. Statistical analysis for (A–F) was conducted by a four‐way ANOVA (*χ* ^2^ test) with Tukey's post hoc comparison. (A) Bar chart of GAG/DNA retained after 10 days of TGF‐*β*3 priming followed by 14 days of culture under different microenvironmental conditions (combinations of oxygen (2% or 5%), pH (6.5, 6.8, or 7.1), glucose (0.5 or 1.0 mM), and osmolarity (350 or 500 mOsm)). 2% oxygen, 7.1 pH, 1.0 mM glucose and 500 mOsm osmolarity (denoted by:@) and 2% oxygen, 6.8 pH, 1.0 mM glucose, 500 mOsm osmolarity (denoted by: €) were significantly increased compared to pH 6.5 groups. (B) Venn diagram displaying *p*‐values of GAG/DNA retained within the bead. Heat mapping highlighted significant effects. pH significantly affected GAG accumulation (*p* = 0.00097). (C) Bar chart showing GAG/DNA released after priming and culture; no significance was observed. (D) Venn diagram of *p*‐values of GAG/DNA released into the media, no significant effects observed. (E) Bar chart showing total GAG/DNA after priming and culture; no significant differences were observed. (F) Venn diagram of *p*‐values of total GAG/DNA (retained + released), showing no significant effects, a trend in glucose was observed (*p* = 0.0769). (G) Aldehyde Fuchsin staining of beads at day 24, across all culture conditions, lighter staining observed in pH 6.5 groups indicates a lower matrix deposition. Scalebar: 200 μm. Full statistical details are provided in Table S3.

**FIGURE 6:**
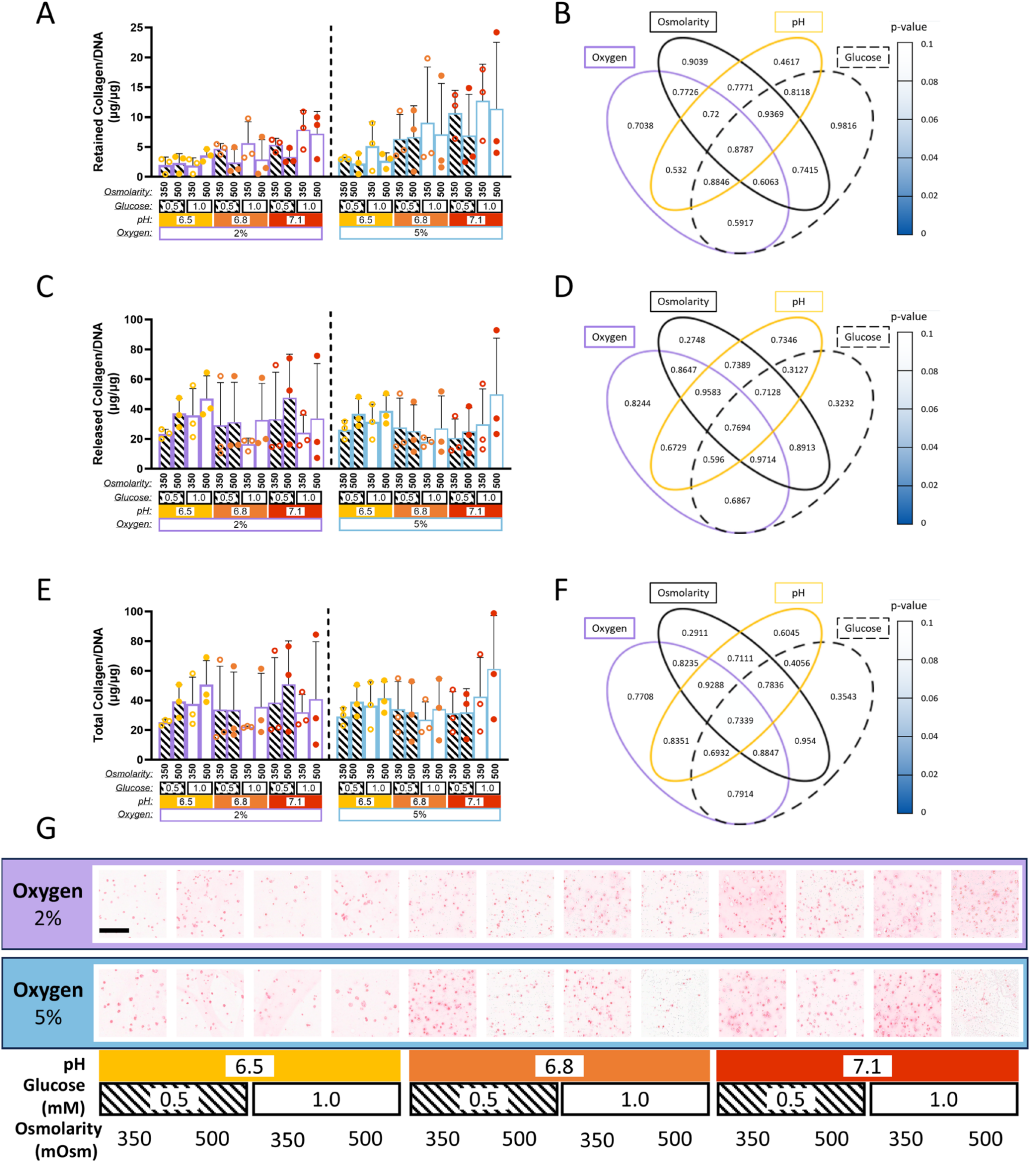
Collagen production of goat NP cells encapsulated in alginate beads subjected to varied microenvironmental conditions. Statistical analysis for (A–F) was conducted by a four‐way ANOVA (*χ* ^2^ test) with Tukey's post hoc comparison. (A) Bar chart of Collagen/DNA retained after 10 days of TGF‐*β*3 priming followed by 14 days of culture under different microenvironmental conditions (combinations of oxygen [2% or 5%], pH [6.5, 6.8, or 7.1], glucose [0.5 or 1.0 mM], and osmolarity [350 or 500 mOsm]). (B) Venn diagram displaying *p*‐values of Collagen/DNA retained within the bead. Heat mapping highlights significant effects and trends. (C) Bar chart showing Collagen/DNA released after priming and culture; no significance was observed. (D) Venn diagram of *p*‐values of Collagen/DNA released into the media, no significant effects observed. (E) Bar chart showing total Collagen/DNA after priming and culture; no significant differences were observed. (F) Venn diagram of *p*‐values of total Collagen/DNA (retained + released). (G) Picrosirius Red staining of beads at day 24, across all culture conditions. Scalebar: 200 μm.

**FIGURE 7:**
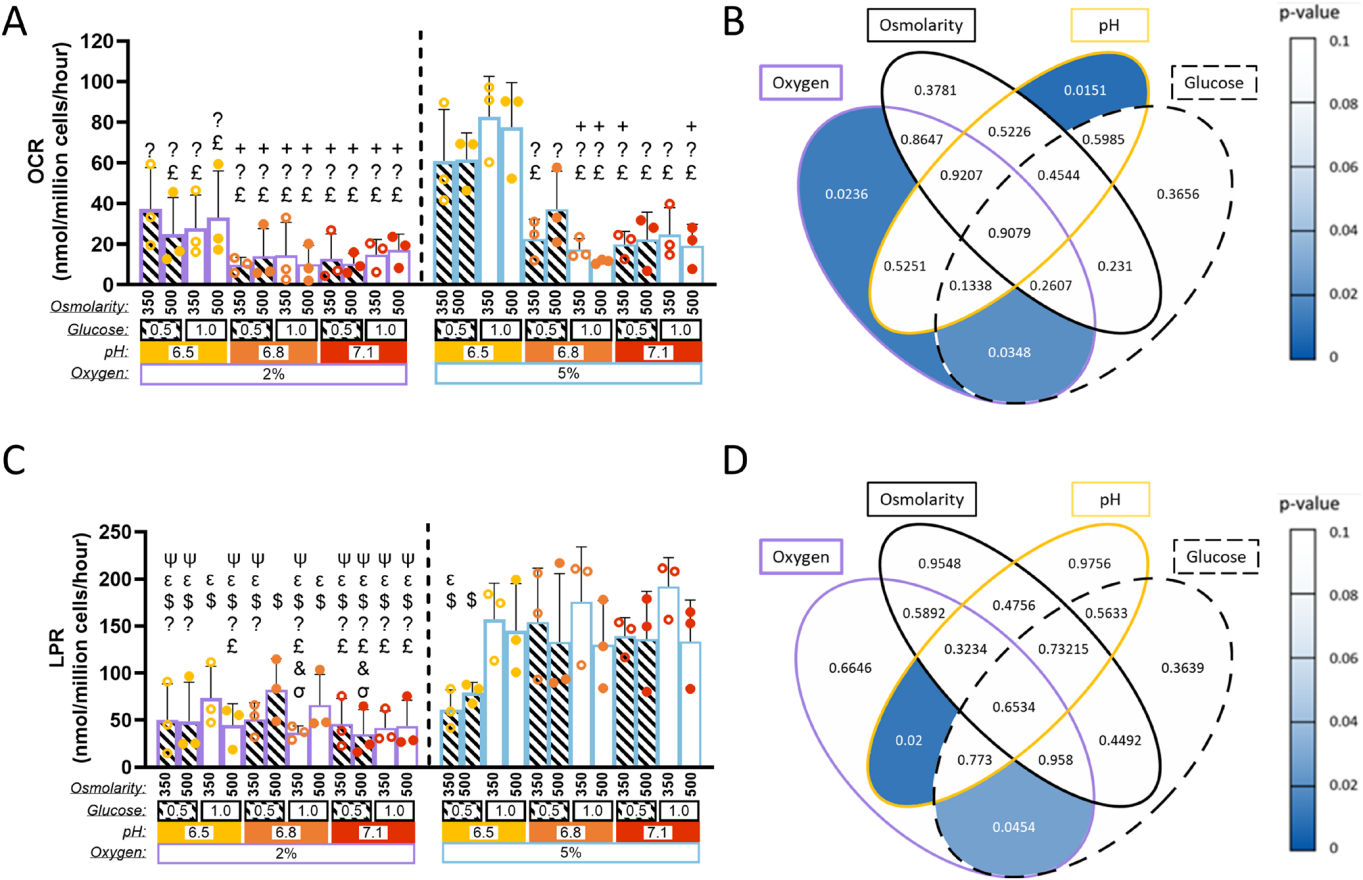
Metabolic rates of goat NP cells encapsulated in alginate beads subjected to varied microenvironmental conditions. Statistical analysis was conducted by a four‐way ANOVA (*χ* ^2^ test) with Tukey's post hoc comparison. (A) Bar chart of oxygen consumption rate (OCR) after 10 days of TGF‐*β*3 priming followed by 3 days of culture under different microenvironmental conditions (combinations of oxygen [2% or 5%], pH [6.5, 6.8, or 7.1], glucose [0.5 or 1.0 mM], and osmolarity [350 or 500 mOsm]). Significance to 5% oxygen pH 6.5 groups denoted by “?” (1.0 mM glucose, 350 mOsm osmolarity), “£” (1.0 mM glucose, 500 mOsm osmolarity), and “+” (0.5 mM glucose, 350 and 500 mOsm osmolarity). (2.45E‐06 ≤ *p* ≤ 0.0414) (B) Venn diagram displaying *p*‐values of OCR. Heat mapping highlights significant effects and trends. (C) Bar chart showing lactate production rate (LPR) released after priming and culture. Significance to 5% groups is denoted by: “*ψ*” (6.8 pH, 0.5 mM glucose, 350 mOsm osmolarity), “*ε*” (6.8 pH, 1.0 mM glucose, 350 mOsm osmolarity), “$” (7.1 pH, 1.0 mM glucose, 350 mOsm osmolarity), “&” (7.1 pH 0.5 mM glucose, 350 mOsm osmolarity), “?” (6.5 pH, 1.0 mM glucose, 350 mOsm osmolarity), “£” (6.5 pH, 1.0 mM glucose, 500 mOsm osmolarity), and “*σ*” (7.1 pH, 0.5 mM glucose, 500 mOsm osmolarity). (D) Venn diagram of *p*‐values of LPR. Full statistical details are provided in Table S4 (OCR) and S5 (LPR).

Figures 3, 4, 5, 6, 7 display quantitative and semi‐quantitative data, analyzed through a four‐way ANOVA with a Type III Wald chi‐square analysis to compare whether each factor (oxygen, glucose, pH, and osmolarity) and interactions between these factors significantly contribute to changes in the cellular response. A Tukey's post hoc analysis was conducted to analyze pairwise comparisons across all combinations (24 groups). Data from these analyses are presented in two ways, via bar chart and Venn diagram.

The bar charts contain 24 bars with each bar representing a unique combination of the four experimental variables. These factors are listed along the x‐axis, grouped by oxygen level (2% [left] and 5% [right]) and further categorized by combinations of glucose, pH, and osmolarity shown in labeled boxes beneath the x‐axis. Bars are color coordinated to complement the x‐axis labeling. Statistical significance indicated on the bar charts by symbols represents the results of Tukey's post hoc analysis.

Venn diagrams illustrate the *p*‐values obtained from the Type III Wald chi‐square analysis. Each oval represents one of the four environmental factors (oxygen, pH, glucose, and osmolarity), while the overlapping regions indicate combinations of these variables. Each region contains a *p*‐value indicating whether the corresponding variable or interaction significantly influences the measured outcome. The Venn diagram is overlaid with a heatmap, where color intensity reflects the strength of influence, with darker blue shades indicating lower (higher significance) *p*‐values.

### 3.2 Microenvironmental pH Plays a Critical Role in Cell Viability

Goat NP cells were encapsulated in alginate beads and primed for 10 days in TGF‐*β*3 to promote the development of a protective ECM niche [24, 30]. Following priming, the beads were exposed to 24 distinct microenvironmental conditions, comprising various combinations of pH, oxygen, glucose, and osmolarity. Live/dead confocal imaging at Day 24 (Figure 3A) showed a marked reduction in calcein staining (live cells) in the pH 6.5 groups compared to pH 6.8 and 7.1. Significant reductions in cell viability were observed at pH 6.5 subjected to 2% oxygen for both glucose and osmolarity levels. This reduction at pH 6.5 was also observed at 5% oxygen, 0.5 mM glucose for both osmolarity levels (7.41E‐12 ≤ *p* ≤ 0.022) (Figure 3B, denoted by !). Significance was also observed at 5% oxygen, pH 6.5, 1.0 mM glucose at both osmolarities when compared to all pH 6.8 and 7.1 groups except for the 2% oxygen, pH 6.8, glucose 0.5 mM, and 500 mOsm group (Figure 3B, denoted by #). Significance was also noted at pH 6.8 and 500 mOsm osmolarity between the 2% oxygen, 0.5 mM glucose group and the 5% oxygen, 1.0 mM glucose group (*p* = 0.033, denoted by *θ*), as well as between the 2% oxygen, pH 6.8, 0.5 mM glucose, 500 mOsm osmolarity group and the 5% oxygen, pH 7.1, 1.0 mM glucose, 350 mOsm Osmolarity group (*p* = 0.014, denoted by &) (Figure 3B). Across all comparisons, changes in pH were consistent, highlighting pH as a key influencing factor. Chi‐squared analysis identified pH as having the most significant impact on cell viability (*p* = < 2.2E‐16) with glucose also showing a highly significant effect (*p* = 0.021). Additionally, a significant interaction was observed between pH and osmolarity (*p* = 0.033) (Figure 3C).

### 3.3 pH Significantly Influences DNA Content, Corroborating Cell Viability Observations

A four‐way ANOVA with Tukey's post hoc comparisons revealed that DNA levels were significantly reduced in groups cultured at pH 6.5, 1.0 mM glucose, and 500 mOsm osmolarity—for both 2% oxygen and 5% oxygen when compared to three other groups. These comparisons are denoted by the symbols &, %, and $ in Figure 4A, and include the following reference groups: (a) %: 2% oxygen, pH 7.1, 1.0 mM glucose, 350 mOsm (*p* = 0.037 and 0.035); (b) &: 5% oxygen, pH 7.1, 0.5 mM glucose, 350 mOsm (*p* = 0.048 and 0.046); (c) $: 5% oxygen, pH 7.1, 1.0 mM glucose, 350 mOsm (*p* = 0.041 and 0.039).

Across all conditions, this trend reflects a consistent decrease in DNA content with a shift from healthy pH (pH 7.1) to degenerated pH (pH 6.5) (Figure 4A). A chi‐squared analysis identified pH as the only significant factor influencing DNA content (*p* = 0.023), while all other variables and interactions were non‐significant. This highlights pH as the most influential factor (Figure B4), consistent with the group comparisons and trends observed in cell viability. Histological analysis using H&E staining further supported these findings, with lighter staining observed in the pH 6.5 groups, indicating lower cellular content and matrix deposition (Figure 4C).

### 3.4 Acidic Microenvironments Inhibit GAG Production

Figure 5 outlines the effect of oxygen, pH, glucose and osmolarity on cellular GAG production. A Tukey's post hoc analysis of retained GAG/DNA, representing GAG content within the alginate bead normalized to DNA content, showed GAG/DNA to be significantly reduced in all 2% oxygen groups at pH 6.5 (*p* = 0.009, 0.016, 0.014 and, 0.023 respectively; denoted by:@, Figure 5A) and when compared to 2% oxygen, pH 7.1, 1.0 mM glucose and 500 mOsm osmolarity. 2% oxygen, pH 7.1, 1.0 mM glucose and 500 mOsm osmolarity was also significantly increased compared to three 5% groups at pH 6.5, (a) 0.5 mM glucose, 350 mOsm osmolarity, 0.5 mM glucose, 500 mOsm osmolarity and, (b) 1.0 mM glucose 350 mOsm osmolarity (*p* = 0.013, 0.017, 0.034 respectively; denoted by@).

Reduced GAG/DNA was also noted at 2% oxygen, pH 6.5, 350 mOsm osmolarity at 0.5 mM and 1.0 mM glucose, as well as 5% oxygen, pH 6.5, 0.5 mM glucose, 350 mOsm osmolarity (*p* = 0.031, 0.044 and, 0.041 respectively; denoted by €) when compared to 2% oxygen, pH 6.8, 1.0 mM glucose, 500 mOsm osmolarity. Across all these comparisons a change in pH from a more acidic 6.5 to neutral 6.8 or 7.1 is consistent, indicating pH as the most influential factor on GAG production. A chi squared analysis corroborated this finding with retained GAG/DNA significantly impacted by decreasing microenvironmental pH (*p* < 0.001; Figure 5B). Similar to DNA, none of the other variables significantly affected the amount of retained GAG/DNA within the alginate beads, suggesting that GAG production is primarily influenced by the microenvironmental pH.

Released GAG/DNA refers to the amount of GAG measured in the culture media normalized to DNA content. Media was collected throughout the culture period, lyophilized and resuspended in ultra‐pure water. No significant differences were noted in GAG/DNA content within the media as a result of oxygen, glucose osmolarity or pH (Figure 5C,D). Total GAG/DNA, representing the sum of retained and released GAG normalized to DNA (Figure 5E,F), was not significantly affected by any of the factors. However, chi squared analysis revealed a trend toward glucose influencing total GAG levels (*p* = 0.076; Figure 5F). Histological staining of the retained GAG within the alginate beads (Figure 5G) further supported the impact of pH on GAG synthesis, revealing reduced retained GAG deposition in the pH 6.5 groups, as evidenced by lighter matrix staining.

### 3.5 Collagen Production Is Maintained Under Variable Microenvironmental Conditions

Collagen/DNA content retained within the alginate bead was not significantly affected by variations in oxygen, pH, glucose, or osmolarity within clinically relevant ranges (Figure 6A,B). Similarly, collagen/DNA levels released into the media remained consistent across all conditions, resulting in no significant differences in total collagen/DNA (Figure 6C–F). Four‐way ANOVA, Chi‐squared analysis, and Tukey's post hoc comparisons confirmed that neither individual factors nor their interactions significantly influenced Collagen/DNA content. These findings are supported by picrosirius red staining, which showed no notable differences between groups (Figure 6G).

### 3.6 Oxygen Concentration and pH Levels Significantly Influence Oxygen Consumption Rate

Metabolic OCR, assessed via seahorse analysis, highlighted the key influence of microenvironmental factors, particularly oxygen and pH, on cellular metabolic activity (Figure 7A,B). A four‐way ANOVA with Tukey's post hoc comparisons identified several significant differences.

Groups marked by the symbol “?” exhibited significantly reduced OCR compared to the reference group cultured at 5% oxygen, pH 6.5, 1.0 mM glucose, and 350 mOsm osmolarity. The groups denoted by “?” include all 2% oxygen groups and all 5% groups at pH 6.8 and 7.1 (0.0000024 ≤ *p* ≤ 0.0133).

Groups denoted by “£” were significantly reduced compared to 5% oxygen, 6.5 pH, 1.0 mM glucose, and 500 mOsm osmolarity. Significantly reduced groups include all other 2% oxygen groups excluding 2% oxygen, 6.5 pH, 0.5 mM glucose and 350 mOsm osmolarity and all 5% oxygen groups at pH 6.8 and 7.1 excluding 5% oxygen, 6.8 pH, 0.5 mM glucose, and 500 mOsm osmolarity (0.000014 ≤ *p* ≤ 0.0177).

Groups denoted by “+” were significantly reduced compared to 5% oxygen, 6.5 pH, 0.5 mM glucose at both osmolarity levels. These groups include all 2% groups at pH 6.8 and 7.1 as well as four additional 5% oxygen groups (a) and (b) 6.8 pH, 1.0 mM glucose at both osmolarities, (c) 7.1 pH, 0.5 mM glucose, 350 mOsm osmolarity and (d) 7.1 pH, 1.0 mM glucose and 500 mOsm osmolarity (0.0021 ≤ *p* ≤ 0.0414).

A chi‐squared analysis was performed to assess the impact of microenvironmental factors on OCR. Significant effects were observed for both oxygen (*p* = 0.023) and pH (*p* = 0.015), indicating that metabolic activity in NP cells is sensitive to both oxygen availability and microenvironmental acidity (Figure 7B). A significant interaction between oxygen and glucose (*p* = 0.034) highlighted that the effect of microenvironmental oxygen levels on cellular OCR is altered by microenvironmental glucose concentration, highlighting the relationship between these two key factors. No significant effects were found for glucose or osmolarity alone, suggesting that OCR is primarily regulated by microenvironmental oxygen and pH. However, glucose appeared to influence OCR when combined with microenvironmental oxygen concentrations.

### 3.7 Oxygen Dependent Microenvironmental Interactions Modulate Cellular Lactate Production Rates

Metabolic LPR was quantified simultaneously to OCR to assess the impact of microenvironmental factors on glycolytic activity (Figure 7C,D). A four‐way ANOVA was performed to determine significance between groups (Figure 7C). LPR was significantly reduced in groups denoted by “$” when compared to the reference group cultured at 5% oxygen, pH 7.1, 1.0 mM glucose, and 350 mOsm osmolarity (Figure 7A). Significantly reduced groups include all 2% oxygen groups and 5% oxygen groups at pH 6.5, 0.5 mM glucose at both osmolarities (4.35E‐05 ≤ *p* ≤ 0.0156).

Similarly, groups marked with “*ε*” were significantly reduced compared to the group at 5% oxygen, pH 6.8, 1.0 mM glucose, and 350 mOsm osmolarity. Significantly reduced groups include 5% oxygen, pH 6.5, 0.5 mM glucose, and 500 mOsm osmolarity as well as all 2% oxygen groups excluding the group at pH 6.8, 1.0 mM glucose, and 500 mOsm osmolarity (0.0004 ≤ *p* ≤ 0.0356).

The “*ψ*” and “?” symbols indicate significant reductions relative to two 5% oxygen groups: (a) pH 6.8, 0.5 mM glucose, and 350 mOsm osmolarity and (b) pH 6.5, 1.0 mM glucose, 350 mOsm osmolarity. All significantly reduced groups under these comparisons were cultured at 2% oxygen, including: (a, b) pH 6.5, 0.5 mM glucose, at both osmolarities, (c) pH 6.5, 1.0 mM glucose at 500 mOsm osmolarity, (d, e) pH 6.8, 350 mOsm osmolarity, at 0.5 mM and 1.0 mM glucose, and all 7.1 pH groups (0.0037 ≤ *p* ≤ 0.0316).

The “&” symbol denotes groups significantly reduced compared to 5% oxygen, pH 7.1, 0.5 mM glucose, 350 mOsm. These include two 2% oxygen groups: (a) pH 6.8, 1.0 mM glucose, and 350 mOsm osmolarity (*p* = 0.034) and (b) pH 7.1, 0.5 mM glucose, and 500 mOsm osmolarity (*p* = 0.029).

Groups marked with “£” showed significant reductions relative to 5% oxygen, pH 6.5, 1.0 mM glucose, and 500 mOsm osmolarity. These comparisons involve 2% oxygen groups at 1.0 mM glucose and 500 mOsm, at pH 6.5 and 6.8, as well as all 2% oxygen groups at pH 7.1 (0.0151 ≤ *p* ≤ 0.0474).

Finally, groups marked with “*σ*” were significantly reduced compared to 5% oxygen, pH 7.1, 0.5 mM glucose, 500 mOsm osmolarity. This includes two 2% oxygen conditions: (a) pH 6.8, 1.0 mM glucose, and 350 mOsm osmolarity (*p* = 0.047) and (b) pH 7.1, 0.5 mM glucose, and 500 mOsm osmolarity (*p* = 0.04) (Figure 7C).

These comparisons highlight that increased oxygen availability in combination with higher pH and glucose levels enhances LPR. Type III Wald chi‐square analysis corroborated these findings showing that although no individual factor significantly altered the LPR on its own, two two‐way interactions were significant; oxygen coupled with glucose (*p* = 0.045), and oxygen coupled with pH (*p* = 0.02) (Figure 7D). These findings indicate that microenvironmental oxygen modulates LPR with its' effect varying based on both glucose availability and pH.

### 3.8 In Silico Modeling Demonstrates How Microenvironmental Changes Can Impact Nutrient Gradients and GAG Accumulation

**FIGURE 8:**
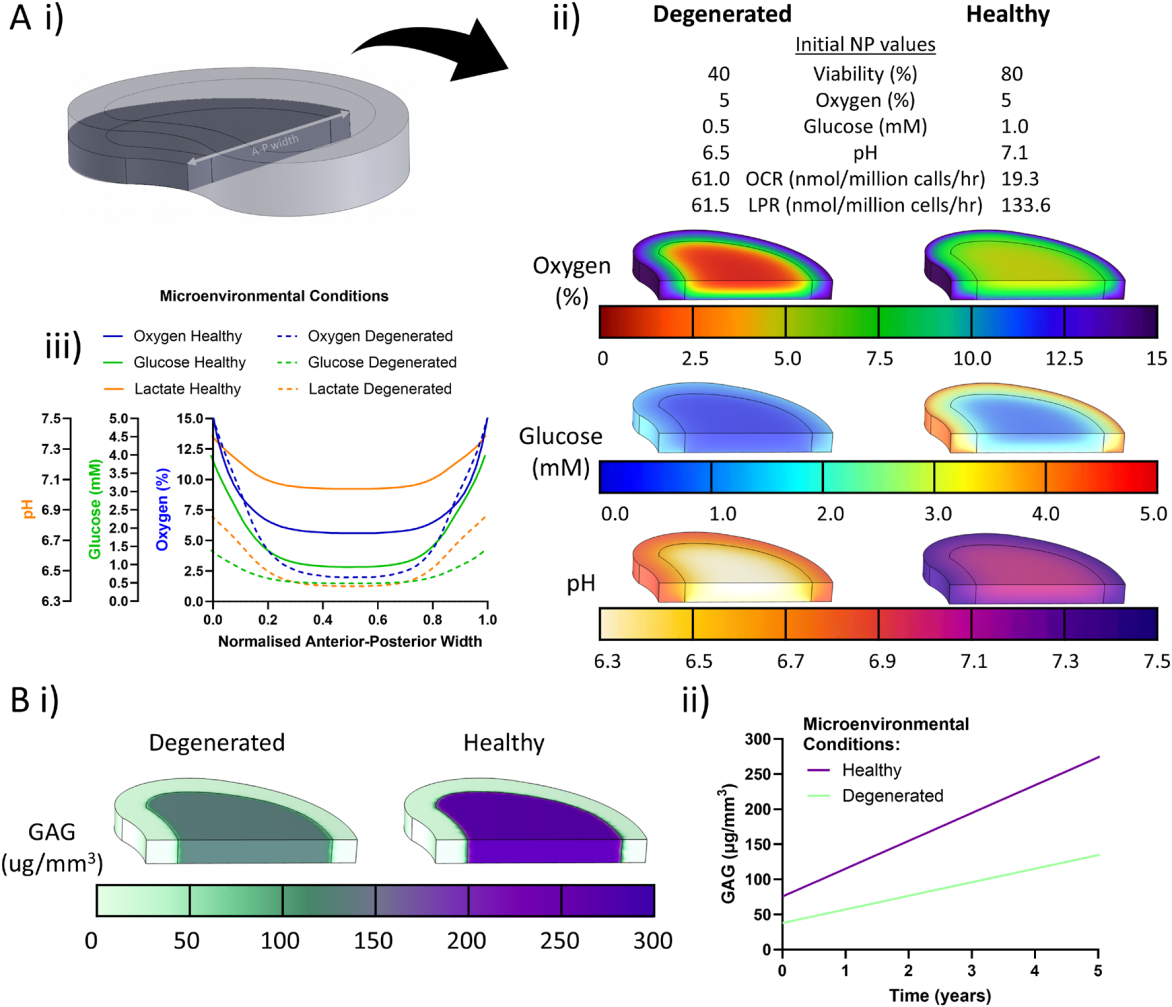
In silico modeling of nutrient microenvironments and GAG synthesis over time based on experimentally derived metabolic and biosynthetic rates. (A) Predicted nutrient microenvironments across a full‐sized goat intervertebral disc, simulated using measured metabolic consumption and production rates. Models reflect steady‐state gradients formed under varying physiological and degenerative boundary conditions. (i) Idealized geometry of goat IVD highlighting the representative quadrant of the geometry used in the model, and anterior–posterior width (A‐P width) used to profile concentrations in (iii). (ii) Predicted oxygen (top), glucose (middle), and pH (bottom) distributions across degenerated and healthy groups. (iii) Anterior to posterior profile for pH, glucose and oxygen at mid height through the idealized IVD. (B) Predicted GAG as a result of healthy and degenerated NP microenvironments. (i) heatmaps highlighting the difference in GAG in the NP region at a 5‐year timepoint. (ii) Graph showing the divergence between healthy and degenerated GAG accumulation within the NP over 5 years.

Figure 8 outlines the results from the in silico modeling of an idealized goat disc under healthy and degenerated states at 5% oxygen. Modeling was conducted on a disc quadrant as outlined in Figure 8A,i. In the degenerated state, central oxygen concentrations dropped to 2.5% as a result of elevated OCR despite boundary conditions being maintained the same as the healthy state, reflecting the 5% oxygen microenvironments chosen for each state. Glucose concentrations in the degenerated state showed a more uniform but reduced gradient across the disc, with the central region of the NP measuring approximately 0.5 mM. In contrast, pH values in the degenerated state were reduced when compared to the healthy state. This is primarily contributed to altered boundary conditions in the degenerated state and increased LPRs. (Figure 8A,ii). Normalized anterior–posterior measurements are shown in Figure 8A,iii for both healthy and degenerated profiles, mapped across the mid‐section of the disc as outlined in Figure 8A,i. These profiles highlight the difference in concentration and gradients across the disc for each species between healthy and degenerated states.

GAG profiles corresponding to matrix production as a result of healthy and degenerated microenvironments are outlined in Figure 8B. Profiles at a 5‐year timepoint reveal a substantial reduction in GAG in the degenerated condition, attributed to decreased cell viability under hostile microenvironmental conditions (Figure 8i). Trajectories of GAG within the NP region are outlined in Figure 8ii. Healthy microenvironmental conditions resulted in a 10‐year accumulation of 475 μg/mm^3^ with a slope of 39.63 while degenerated conditions resulted in a 10‐year accumulation of 232 μg/mm^3^ with a slope of 19.35, indicating the increasing divergence between healthy and degenerated states over time.

## 4 Discussion

The primary goal of this study was to investigate how variations in microenvironmental factors in combination affect the matrix production and metabolism of caprine NP cells in a 3D alginate culture model. Matrix synthesis and metabolic rates were analyzed as a function of microenvironmental oxygen, glucose, pH, and osmolarity correlating to those observed in both healthy and degenerate IVDs. pH was identified as the most influential factor across all measured parameters, significantly impacting cell viability, DNA content, GAG synthesis, and metabolic activity. Secondary to pH, oxygen was the next most impactful factor, with its greatest influence noted in changes to metabolic rates. While glucose and osmolarity had more nuanced effects, their contributions became significant when interacting with other factors. Although this study focused on caprine cells, the general trends observed are consistent with reports from human NP cells under similar conditions, suggesting conserved microenvironmental sensitivities across species. These results indicate that low pH microenvironments are highly detrimental to NP cell function, with neutral pH levels in combination with sufficient oxygen and glucose availability supporting optimal cellular behavior.

The most consistent observation was the negative impact pH 6.5 had on NP cell viability, DNA content, and matrix synthesis, indicating that critically low pH directly limits NP cell survival and matrix production, creating an inhospitable environment even when nutrients and oxygen are present. This aligns with previous studies demonstrating that acidic pH environments lead to reduced viability in NP cells [30, 32, 35, 50]. Furthermore, histological staining of H&E confirmed reduced cellular content and matrix in the low pH groups, suggesting that pH impacts both cell survival and matrix production. Statistical analysis was repeated excluding the pH 6.5 group to determine whether the strong effect of low pH masked the impact of other microenvironmental factors as the median pH in previous studies has been reported to fall at 7.0, however, no additional significant differences between groups were observed. In advanced IVD degeneration, where the presence of these low pH microenvironments is more common, the effect of pH on NP matrix production highlights this cohort to be less amenable to regenerative therapies. As goat NP cells are derived from healthy, non‐degenerate tissue, the observed sensitivity to pH may even underestimate the resilience of human cells that have adapted to chronic degenerative conditions.

Consistent with the viability and DNA results, retained GAG/DNA content was significantly lower in groups cultured at pH 6.5, a response previously linked to diminished cell viability and suppressed GAG synthesis under acidic conditions [30, 51]. These findings suggest that GAG synthesis is highly sensitive to reduced pH microenvironments. Released and total GAG/DNA data did not differ significantly across the microenvironmental conditions. However, a glucose‐dependent trend suggested that glucose may play a secondary role in matrix synthesis consistent with previous studies. Bibby and Urban found that glucose levels below 0.5 mM compromised cell viability and matrix synthesis [27]. As glucose is the primary energy source for NP cells that rely on anaerobic glycolysis, a reduction in this nutrient limits cellular energy resulting in reduced GAG synthesis and cellular function [29]. Neidlinger‐Wilke et al. also demonstrated that reducing glucose levels led to decreased GAG production [52]. This study builds on these findings by showing that glucose effects become particularly important when combined with low pH, highlighting the interdependence of nutrient availability and pH balance in determining matrix outcomes.

While low pH microenvironments severely limited GAG synthesis, collagen production was not significantly altered by the range of microenvironmental factors. Collagen/DNA levels remained consistent, regardless of oxygen and glucose concentrations, osmolarity, or pH. This resilience may reflect the inherently slow turnover of collagen in disc tissue compared to proteoglycans, meaning that short‐term insults are less likely to alter collagen output. This effect has been previously observed where at low pH levels (< 6.5) DNA content was decreased while COL1 and COL2 expression remained unchanged [35]. Another study cultured bovine NP cells in 3D agarose constructs. Oxygen levels between the groups were varied between 20% and 2% with no significant differences observed in collagen/DNA at the 42‐day timepoint [53]. O'Connell et al. analyzed the effect of osmolarity on 2D and 3D NP cell cultures at 300, 400, and 500 mOsm and noted no significant differences in collagen production at day 28 (2D) or 42 (3D) [54]. Previous work from our laboratory demonstrated that pH strongly influences the Collagen/DNA content produced by bone marrow stem cells (BMSCs) particularly at pH 6.5 compared to 7.1. In contrast, articular chondrocytes (AC) assessed under the same conditions in the same study did not show a significant response [30]. This effect was again observed in AC under normoxic and hypoxic conditions with no significance in collagen/DNA observed as a function of oxygen [55]. NP cells have been shown to have a similar phenotype to AC cells with similar morphologies and overlapping ECM markers [56, 57, 58] suggesting collagen production within these cell types may be more resilient to short‐term microenvironmental changes but could still be affected with long‐term exposure. Collagen turnover rates have been shown to be disproportionately lower than those of proteoglycans [59], with collagen turnover being reported at 0.52% per year while GAGs exhibit rates more than tenfold higher at 8.6% per year [60, 61]. This highlights the importance of maintaining proteoglycan presence within the disc to preserve IVD function in the short term, while collagen stability ensures long‐term tissue integrity. To further clarify functional implications, future studies could incorporate mechanical testing or gene expression analyses for key matrix markers to complement histological findings.

Seahorse analysis revealed that both OCR and LPR were significantly affected by both oxygen and pH, emphasizing their critical role in NP cell metabolic activity. A Pasteur effect is defined as the suppression of anaerobic glycolysis in the presence of oxygen, meaning when oxygen availability is reduced cells have a greater affinity for glycolysis, reducing OCR while increasing glucose consumption rate (GCR) and LPR. Conversely, when oxygen concentration increases, cells rely on aerobic respiration, increasing OCR while reducing GCR and LPR [62]. This effect was observed for OCR when oxygen availability was higher (5% oxygen), and pH was reduced to 6.5. Increased OCR observed in degenerated microenvironments may result from this Pasteur effect, as low pH inhibits glycolytic enzymes and reduces glycolytic efficiency, forcing NP cells to depend more on oxidative phosphorylation for energy [47]. LPR increased at higher oxygen concentrations, converse to expectations from the Pasteur effect, except for low glucose groups at pH 6.5 and 5% oxygen where cells become more reliant on oxygen as a source of energy. Several previous studies have observed a positive Pasteur effect in NP cells with increasing oxygen leading to higher OCR and reduced LPR [63, 64]. These effects were not fully replicated in our study, likely due to cellular insults being applied in combination rather than on an individual basis. Previous studies were cultured in 5.55 mM glucose at pH 7.4, except in cases where these were the variable in question, whereas in our culture groups all glucose and pH levels were reduced in comparison. Therefore, as oxygen availability was increased from 2% to 5% it is possible the cells may have become more metabolically active overall, leading to an increased LPR at 5% in most groups. Although varying glucose alone did not have a significant impact, its interactions with oxygen and pH significantly altered both OCR and LPR. These findings align with the known reliance of NP cells on glycolysis under hypoxic conditions [65] and further suggest that oxygen concentration and neutral pH are necessary to support healthy cellular metabolism. This reinforces that NP metabolism is governed by the interplay of microenvironmental signals rather than by any single cue, with glycolysis dominant under hypoxia but oxygen and neutral pH required to sustain efficient energy production.

An in silico model of a goat IVD was used to further explore the magnitude of these findings at the whole tissue level. Due to the lack of variance observed in metabolic rates at 2% oxygen, healthy and degenerated microenvironments were simulated at 5% oxygen. Experimentally determined changes in OCR, LPR, and viability were applied to the models revealing that under identical oxygen boundary conditions, central disc oxygen levels declined sharply to 2.5% in the degenerated state due to increased consumption levels. In silico modeling has previously shown that degenerative changes in the disc create oxygen gradients, leading to reduced oxygen availability in the central region [13, 17, 27]. In this study, changes in OCR driven by microenvironmental factors directly altered the oxygen profile within the disc, suggesting that such shifts in cellular behavior may contribute to the progression of degenerative changes in the intradiscal microenvironment. Glucose levels in the degenerated state were also depleted across the whole disc. To date, profiling of glucose in IVD tissue has relied heavily on in silico modeling across many species [66, 67]. This study complements modeling of glucose in the IVD highlighting the impact perturbations in cellular metabolic rates can have on nutrient availability within the disc thereby providing mechanistic links between cell‐scale responses and tissue‐scale degeneration. pH was considerably reduced in the degenerated state, illustrating the whole disc effect that can be observed at such high lactate levels. While the current model treated lactate primarily as a metabolic by‐product contributing to acidification, it is recognized that AF cells may also utilize lactate as an alternative energy source [27], which could influence local pH dynamics and warrants consideration in future model refinements. Since neutral pH is essential for cellular function and viability, the elevated lactate levels in the IVD would present a major challenge for the success of cell‐based therapies in vivo, leading to reduced cell viability and less favorable metabolic activity [68, 69, 70]. This emphasizes the difficulty of achieving therapeutic success in acidic, nutrient‐depleted discs, where transplanted cells would likely face hostile metabolic conditions.

GAG accumulation within the degenerated disc was substantially reduced compared to the healthy model when viability and GAG synthesis rates were projected over a 5‐year timescale. This finding complements the differences observed in GAG content between healthy and degenerated NP tissue from previous in vivo studies. Gullbrand et al. induced degeneration in a goat in vivo model through injection of chondroitinase ABC highlighting the decrease in GAG visible from alcian blue staining after 12 weeks post injection [28]. Yin et al. blocked nutritional and metabolite diffusion to the IVD in a goat model by sealing the CEP with cement, resulting in reduced GAG accumulation after 48 weeks compared to controls [71]. These changes have also been observed in human, bovine, and ovine discs [72, 73, 74, 75] highlighting the importance of both microenvironmental changes and GAG synthesis over time. Simulations from this study illustrate that even minor shifts in microenvironmental parameters can trigger cascading effects on cellular metabolism and matrix synthesis, ultimately leading to long‐term consequences for IVD tissues at the whole organ level. Modeling these cellular changes across the entire disc helps bridge the gap between cellular behavior and tissue‐level outcomes, providing a powerful framework for predicting the trajectory of degenerative changes and for evaluating the potential efficacy of regenerative therapies.

While many studies have examined the individual effects of oxygen, osmolarity, glucose, and pH on NP cell behavior, a key strength of this study lies in the experimental design, which integrates these factors to capture their combined influence in a way that closely reflects in vivo conditions. This approach enabled the identification of interactions between factors as well as their individual effects. These interactions highlight that NP cell responses cannot be fully understood by examining individual factors in isolation, and that a multifactorial approach is essential for elucidating how cells respond to the complex IVD microenvironment. The chi squared analyses highlighted the interdependence of these microenvironmental factors, and the effect they can have on cellular behavior that is not observed when these factors are analyzed individually. Therefore, experimental culture methods should reflect the complex nature of the IVD microenvironment to ensure changes in cellular behavior are fully understood when designing cellular therapies.

Additionally, embedding NP cells in a 3D alginate hydrogel combined with priming more closely replicates the native extracellular matrix, supporting restoration of the cell phenotype and spatial organization compared to 2D culture methods [76, 77]. while also providing diffusion characteristics that better mimic in vivo conditions [36, 78]. All groups were pretreated with TGF‐*β3* under identical conditions; therefore, the differences observed are attributed to the imposed microenvironmental factors rather than to TGF‐*β3* withdrawal. Nevertheless, potential effects of TGF‐*β* withdrawal on NP cell activity warrant further investigation. Many studies have demonstrated that degeneration of the IVD microenvironment is not linear with respect to degeneration grade, with large variations observed in cellular and environmental measurements [16, 18, 79, 80]. This study enabled a high‐throughput analysis of 24 microenvironmental combinations, accounting for all microenvironmental variants in the studied factors. Understanding the effect of these combinations is important to determine the broader implications of microenvironmental changes. However, several limitations should be acknowledged. First, the study was conducted over a 24‐day culture period, with 14 days of microenvironmental insult, which may not capture longer‐term matrix remodeling or phenotypic shifts as observed in vivo where other key factors such as cytokines and ECM interactions must be considered [81, 82, 83]. Secondly, although alginate facilitates cell culture in an accessible 3D structure, due to its inert properties it lacks cell–matrix adhesion cues which are known to influence NP cell behavior [78, 84, 85, 86]. Although brief exposure to atmospheric oxygen during handling could have induced transient oxidative stress, these effects were minimized by consistent handling and uniformity across groups and the use of a hypoxic chamber for Seahorse metabolic analysis. Additionally, the goat cells used in this study were isolated from healthy discs, whereas human patients targeted for regenerative treatments typically present with mild degeneration. As a result, their cells may already exhibit adaptive responses to the altered microenvironment of the degenerated disc. The absence of dynamic mechanical loading, a key regulator of nutrient transport and mechanotransduction in the IVD [87, 88], represents another limitation. Future studies employing bioreactor or explant systems could help clarify how loading interacts with microenvironmental cues to influence NP cell behavior. Lastly, while goat NP cells serve as a useful alternative to human cells, species‐specific differences may limit the direct translation of these results to human applications. The geometry of the goat IVD is comparable to that of humans when scaled to species size [28]. In addition, similar ECM properties such as diffusion coefficients and hydration create a microenvironment within the disc that closely resembles that of the human IVD [36, 89, 90]. However, OCR in human cells has been reported to be lower than that of goat cells. McDonnell et al. reported OCR for goat NP cells cultured in 5.55 mM glucose to be approximately 3.8 (nmol/million cells/h) falling to approximately 1.0 (nmol/million cells/h) in human cells [17], supporting previous findings that IVD cells obtain most of their energy through glycolysis [21, 63]. Therefore, the impact of microenvironmental changes on alterations within the human IVD may be less pronounced than observed in the in silico models in this study. However, analysis in human cells at lower glucose levels is needed to determine how OCRs would be affected. These limitations are common to many in vitro disc models, and the insights gained from this study offer valuable direction for future research. Future work incorporating human NP cells, or organ‐level cultures, would help confirm whether the trends observed in the caprine model translate to the human degenerative setting.

In summary, this study demonstrates that microenvironmental pH is the dominant factor regulating caprine NP cell viability, matrix production, and metabolic activity, followed by oxygen concentration and specific interactions with glucose availability. By integrating factorial cell culture with in silico modeling, we have successfully linked nutrient microenvironmental factors to tissue‐level consequences, offering insight into why degenerated discs are challenging targets for cell‐based regenerative therapies. Understanding these relationships provides a foundation for the development of regenerative therapies aimed at restoring a more physiological microenvironment in the degenerative IVD while also serving as a critical knowledge base for the effective application of translational animal models in treatment design. Importantly, our findings also demonstrate that interactions between microenvironmental factors influence NP cell behavior more profoundly than isolated effects, reinforcing the need to study these variables in combination.

## 5 Conclusion

This study demonstrates that pH is the dominant regulator of caprine NP cell viability, matrix production and metabolic rates, with oxygen and glucose also noted to play an influential role. Interactions between these variables have also proven to be influential on viability and metabolism, highlighting the importance of studying these factors in combination. Applying the outcomes of this study in a 3D full disc in silico model demonstrates how small changes in the disc microenvironment can significantly influence local microenvironments and long‐term matrix deposition. This study emphasizes the importance of accurately mimicking the native IVD microenvironment in vivo when developing and evaluating regenerative therapies, providing valuable insights into the conditions required to sustain viability and matrix synthesis in a degenerating disc.

## Author Contributions

Niamh Wilson and Conor T. Buckley contributed substantially to the conception and design of the work. Niamh Wilson performed the acquisition and interpretation of literature, data analysis, computational modeling, presentation and interpretation of results, drafting of the article, revising it critically, and final approval. Tara Ní Néill, Jake McDonnell, and Emily McDonnell contributed to the acquisition of laboratory data. Pieter A.J. Brama contributed to the acquisition of animal samples. Conor T. Buckley, as the overall project funding holder, takes responsibility for the integrity of the work from inception to finalized article, and provided substantial contribution to data interpretation and presentation. Niamh Wilson and Conor T. Buckley drafted the manuscript. All authors critically revised the manuscript and approved the final version.

## Funding

This work was supported by the European Research Council, ERC‐2019‐CoG‐864104; INTEGRATE.

## Conflicts of Interest

Conor T. Buckley is an Editorial Board member of JOR Spine and co‐author of this article. He was excluded from editorial decision‐making related to the acceptance of this article for publication in the journal. The remaining authors declare no conflicts of interest.

## Supporting information


**Table S1:** Significant *p*‐values of Tukey's post hoc comparison of viability of goat NP cells encapsulated in alginate beads subjected to various microenvironmental conditions From Figure 1: “!” indicates significant reduction in viability compared to all pH 6.8 and 7.1 groups. “#” indicates significant reduction compared to all pH 6.8 and 7.1 groups excluding the 2% oxygen, pH 6.8, glucose 0.5 mM, and 500 mOsm group.
**Table S2:** Significant *p*‐values of Tukey's post hoc comparison of DNA of goat NP cells encapsulated in alginate beads subjected to various microenvironmental conditions.
**Table S3:** Significant *p*‐values of Tukey's post hoc comparison of Retained GAG/DNADNA of goat NP cells encapsulated in alginate beads subjected to various microenvironmental conditions.
**Table S4:** Significant *p*‐values of Tukey's post hoc comparison of Oxygen Consumption Rates of goat NP cells encapsulated in alginate beads subjected to various microenvironmental conditions.
**Table S5:** Significant *p*‐values of Tukey's post hoc comparison of Lactate Production Rates of goat NP cells encapsulated in alginate beads subjected to various microenvironmental conditions.

## Data Availability

The data that support the findings of this study are openly available on Zenodo at 10.5281/zenodo.16963012.
