# Supplementary material for: Influence of the Intervertebral Disc Microenvironment on Matrix Synthesis and Metabolism in Goat Nucleus Pulposus Cells
Source: JOR Spine. 2026 Jan 14;9(1):e70160. doi: 10.1002/jsp2.70160 (PMC12801399; doi:10.1002/jsp2.70160)
Supplement: Supplementary file 1 — Table S1: Significant p‐values of Tukey's post hoc comparison of viability of goat NP cells encapsulated in alginate beads subjected to various microenvironmental conditions From Figure 1: “!” indicates significant reduction in viability compared to all pH 6.8 and 7.1 groups. “#” indicates significant reduction compared to all pH 6.8 and 7.1 groups excluding the 2% oxygen, pH 6.8, glucose 0.5 mM, and 500 mOsm group. Table S2: Significant p‐values of Tukey's post hoc comparison of DNA of goat NP cells encapsulated in alginate beads subjected to various microenvironmental conditions. Table S3: Significant p‐values of Tukey's post hoc comparison of Retained GAG/DNADNA of goat NP cells encapsulated in alginate beads subjected to various microenvironmental conditions. Table S4: Significant p‐values of Tukey's post hoc comparison of Oxygen Consumption Rates of goat NP cells encapsulated in alginate beads subjected to various microenvironmental conditions. Table S5: Significant p‐values of Tukey's post hoc comparison of Lactate Production Rates of goat NP cells encapsulated in alginate beads subjected to various microenvironmental conditions. [file JSP2-9-e70160-s001.docx]

Data Availability: Supplementary datasets associated with this article can be found in an online repository at DOI: 10.5281/zenodo.16963012

Table S1. Significant p-values of Tukey’s post-hoc comparison of viability of goat NP cells encapsulated in alginate beads subjected to various microenvironmental conditions

From Figure 1: “!” indicates significant reduction in viability compared to all pH 6.8 and 7.1 groups. “#” indicates significant reduction compared to all pH 6.8 and 7.1 groups excluding the 2% oxygen, pH 6.8, glucose 0.5 mM, and 500 mOsm group.

| Comparison denoted by | Microenvironmental group 1 | | | | compared to: | | Microenvironmental group 2 | | | |  |  |
| --- | --- | --- | --- | --- | --- | --- | --- | --- | --- | --- | --- | --- |
|  | Oxygen (%) | pH | Glucose (mM) | Osmolarity (mOsm) | | Oxygen (%) | | pH | Glucose (mM) | Osmolarity (mOsm) | p-value | Significance |
| ! | 2% | 6.5 | 0.5 | 350 | | 2% | | 6.8 | 0.5 | 350 | 2.64E-09 | **** |
|  |  |  |  |  |  |  |  |  |  | 500 | 1.16E-05 | **** |
|  |  |  |  |  |  |  |  |  | 1.0 | 350 | 3.94E-08 | **** |
|  |  |  |  |  |  |  |  |  |  | 500 | 9.82E-09 | **** |
|  |  |  |  |  |  |  |  | 7.1 | 0.5 | 350 | 1.91E-08 | **** |
|  |  |  |  |  |  |  |  |  |  | 500 | 7.62E-10 | **** |
|  |  |  |  |  |  |  |  |  | 1.0 | 350 | 1.84E-10 | **** |
|  |  |  |  |  |  |  |  |  |  | 500 | 1.53E-09 | **** |
|  |  |  |  |  |  | 5% | | 6.8 | 0.5 | 350 | 3.24E-11 | **** |
|  |  |  |  |  |  |  |  |  |  | 500 | 1.42E-10 | **** |
|  |  |  |  |  |  |  |  |  | 1.0 | 350 | 4.50E-08 | **** |
|  |  |  |  |  |  |  |  |  |  | 500 | 1.95E-11 | **** |
|  |  |  |  |  |  |  |  | 7.1 | 0.5 | 350 | 2.02E-10 | **** |
|  |  |  |  |  |  |  |  |  |  | 500 | 4.25E-09 | **** |
|  |  |  |  |  |  |  |  |  | 1.0 | 350 | 7.41E-12 | **** |
|  |  |  |  |  |  |  |  |  |  | 500 | 1.55E-08 | **** |
| ! | 2% | 6.5 | 0.5 | 500 | | 2% | | 6.8 | 0.5 | 350 | 7.52E-09 | **** |
|  |  |  |  |  |  |  |  |  |  | 500 | 3.41E-05 | **** |
|  |  |  |  |  |  |  |  |  | 1.0 | 350 | 1.15E-07 | **** |
|  |  |  |  |  |  |  |  |  |  | 500 | 2.83E-08 | **** |
| ! | 2% | 6.5 | 0.5 | 500 | | 2% | | 7.1 | 0.5 | 350 | 5.53E-08 | **** |
|  |  |  |  |  |  |  |  |  |  | 500 | 2.14E-09 | **** |
|  |  |  |  |  |  |  |  |  | 1.0 | 350 | 5.11E-10 | **** |
|  |  |  |  |  |  |  |  |  |  | 500 | 4.33E-09 | **** |
| ! | 2% | 6.5 | 0.5 | 500 | | 5% | | 6.8 | 0.5 | 350 | 8.88E-11 | **** |
|  |  |  |  |  |  |  |  |  |  | 500 | 3.92E-10 | **** |
|  |  |  |  |  |  |  |  |  | 1.0 | 350 | 1.31E-07 | **** |
|  |  |  |  |  |  |  |  |  |  | 500 | 5.36E-11 | **** |
|  |  |  |  |  |  |  |  | 7.1 | 0.5 | 350 | 5.62E-10 | **** |
|  |  |  |  |  |  |  |  |  |  | 500 | 1.22E-08 | **** |
|  |  |  |  |  |  |  |  |  | 1.0 | 350 | 2.07E-11 | **** |
|  |  |  |  |  |  |  |  |  |  | 500 | 4.48E-08 | **** |
| ! | 2% | 6.5 | 1.0 | 350 | | 2% | | 6.8 | 0.5 | 350 | 6.24E-06 | **** |
|  |  |  |  |  |  |  |  |  |  | 500 | 0.01923 | * |
|  |  |  |  |  |  |  |  |  | 1.0 | 350 | 9.78E-05 | **** |
|  |  |  |  |  |  |  |  |  |  | 500 | 2.41E-05 | **** |
|  |  |  |  |  |  |  |  | 7.1 | 0.5 | 350 | 4.73E-05 | **** |
|  |  |  |  |  |  |  |  |  |  | 500 | 1.70E-06 | **** |
|  |  |  |  |  |  |  |  |  | 1.0 | 350 | 3.80E-07 | **** |
|  |  |  |  |  |  |  |  |  |  | 500 | 3.53E-06 | **** |
|  |  |  |  |  |  | 5% | | 6.8 | 0.5 | 350 | 5.99E-08 | **** |
|  |  |  |  |  |  |  |  |  |  | 500 | 2.88E-07 | **** |
|  |  |  |  |  |  |  |  |  | 1.0 | 350 | 0.000112 | *** |
|  |  |  |  |  |  |  |  |  |  | 500 | 3.52E-08 | **** |
|  |  |  |  |  |  |  |  | 7.1 | 0.5 | 350 | 4.20E-07 | **** |
|  |  |  |  |  |  |  |  |  |  | 500 | 1.02E-05 | **** |
| ! | 2% | 6.5 | 1.0 | 350 | | 5% | | 7.1 | 1.0 | 350 | 1.29E-08 | **** |
|  |  |  |  |  |  |  |  |  |  | 500 | 3.83E-05 | **** |
| ! | 2% | 6.5 | 1.0 | 500 | | 2% | | 6.8 | 0.5 | 350 | 7.71E-06 | **** |
|  |  |  |  |  |  |  |  |  |  | 500 | 0.022905 | * |
|  |  |  |  |  |  | 2% | | 6.8 | 1.0 | 350 | 0.00012 | *** |
|  |  |  |  |  |  |  |  |  |  | 500 | 2.97E-05 | **** |
|  |  |  |  |  |  |  |  | 7.1 | 0.5 | 350 | 5.83E-05 | **** |
|  |  |  |  |  |  |  |  |  |  | 500 | 2.10E-06 | **** |
|  |  |  |  |  |  |  |  |  | 1.0 | 350 | 4.69E-07 | **** |
|  |  |  |  |  |  |  |  |  |  | 500 | 4.37E-06 | **** |
|  |  |  |  |  |  | 5% | | 6.8 | 0.5 | 350 | 7.39E-08 | **** |
|  |  |  |  |  |  |  |  |  |  | 500 | 3.55E-07 | **** |
|  |  |  |  |  |  |  |  |  | 1.0 | 350 | 0.000138 | *** |
|  |  |  |  |  |  |  |  |  |  | 500 | 4.33E-08 | **** |
|  |  |  |  |  |  |  |  | 7.1 | 0.5 | 350 | 5.19E-07 | **** |
|  |  |  |  |  |  |  |  |  |  | 500 | 1.26E-05 | **** |
|  |  |  |  |  |  |  |  |  | 1.0 | 350 | 1.59E-08 | **** |
|  |  |  |  |  |  |  |  |  |  | 500 | 4.72E-05 | **** |
| ! | 5% | 6.5 | 0.5 | 350 | | 2% | | 6.8 | 0.5 | 350 | 2.38E-07 | **** |
|  |  |  |  |  |  |  |  |  |  | 500 | 0.001035 | ** |
|  |  |  |  |  |  |  |  |  | 1.0 | 350 | 3.79E-06 | **** |
|  |  |  |  |  |  |  |  |  |  | 500 | 9.20E-07 | **** |
|  |  |  |  |  |  |  |  | 7.1 | 0.5 | 350 | 1.82E-06 | **** |
|  |  |  |  |  |  |  |  |  |  | 500 | 6.58E-08 | **** |
|  |  |  |  |  |  |  |  |  | 1.0 | 350 | 1.50E-08 | **** |
|  |  |  |  |  |  |  |  |  |  | 500 | 1.35E-07 | **** |
| ! | 5% | 6.5 | 0.5 | 350 | | 5% | | 6.8 | 0.5 | 350 | 2.47E-09 | **** |
|  |  |  |  |  |  |  |  |  |  | 500 | 1.14E-08 | **** |
|  |  |  |  |  |  |  |  |  | 1.0 | 350 | 4.35E-06 | **** |
|  |  |  |  |  |  |  |  |  |  | 500 | 1.47E-09 | **** |
|  |  |  |  |  |  |  |  | 7.1 | 0.5 | 350 | 1.66E-08 | **** |
|  |  |  |  |  |  |  |  |  |  | 500 | 3.89E-07 | **** |
|  |  |  |  |  |  |  |  |  | 1.0 | 350 | 5.55E-10 | **** |
|  |  |  |  |  |  |  |  |  |  | 500 | 1.47E-06 | **** |
| ! | 5% | 6.5 | 0.5 | 500 | | 2% | | 6.8 | 0.5 | 350 | 1.65E-07 | **** |
|  |  |  |  |  |  |  |  |  |  | 500 | 0.000729 | *** |
|  |  |  |  |  |  |  |  |  | 1.0 | 350 | 2.62E-06 | **** |
|  |  |  |  |  |  |  |  |  |  | 500 | 6.36E-07 | **** |
|  |  |  |  |  |  |  |  | 7.1 | 0.5 | 350 | 1.25E-06 | **** |
|  |  |  |  |  |  |  |  |  |  | 500 | 4.57E-08 | **** |
|  |  |  |  |  |  |  |  |  | 1.0 | 350 | 1.05E-08 | **** |
|  |  |  |  |  |  |  |  |  |  | 500 | 9.39E-08 | **** |
|  |  |  |  |  |  | 5% | | 6.8 | 0.5 | 350 | 1.73E-09 | **** |
|  |  |  |  |  |  |  |  |  |  | 500 | 7.98E-09 | **** |
|  |  |  |  |  |  |  |  |  | 1.0 | 350 | 3.01E-06 | **** |
|  |  |  |  |  |  |  |  |  |  | 500 | 1.03E-09 | **** |
|  |  |  |  |  |  |  |  | 7.1 | 0.5 | 350 | 1.15E-08 | **** |
|  |  |  |  |  |  |  |  |  |  | 500 | 2.70E-07 | **** |
|  |  |  |  |  |  |  |  |  | 1.0 | 350 | 3.91E-10 | **** |
|  |  |  |  |  |  |  |  |  |  | 500 | 1.01E-06 | **** |
| # | 5% | 6.5 | 1.0 | 350 | | 2% | | 6.8 | 0.5 | 350 | 0.000181 | *** |
|  |  |  |  |  |  |  |  |  | 1.0 | 350 | 0.002529 | ** |
| # | 5% | 6.5 | 1.0 | 350 | | 2% | | 6.8 | 1.0 | 500 | 0.000671 | *** |
|  |  |  |  |  |  |  |  | 7.1 | 0.5 | 350 | 0.001276 | ** |
|  |  |  |  |  |  |  |  |  |  | 500 | 5.06E-05 | **** |
|  |  |  |  |  |  |  |  |  | 1.0 | 350 | 1.14E-05 | **** |
|  |  |  |  |  |  |  |  |  | 1.0 | 500 | 0.000104 | *** |
|  |  |  |  |  |  | 5% | | 6.8 | 0.5 | 350 | 1.77E-06 | **** |
|  |  |  |  |  |  |  |  |  |  | 500 | 8.61E-06 | **** |
|  |  |  |  |  |  |  |  |  | 1.0 | 350 | 0.002868 | ** |
|  |  |  |  |  |  |  |  |  |  | 500 | 1.03E-06 | **** |
|  |  |  |  |  |  |  |  | 7.1 | 0.5 | 350 | 1.26E-05 | **** |
|  |  |  |  |  |  |  |  |  |  | 500 | 0.000293 | *** |
|  |  |  |  |  |  |  |  |  | 1.0 | 350 | 3.72E-07 | **** |
|  |  |  |  |  |  |  |  |  |  | 500 | 0.001044 | ** |
| # | 5% | 6.5 | 1.0 | 500 | | 2% | | 6.8 | 0.5 | 350 | 0.000126 | *** |
|  |  |  |  |  |  |  |  |  | 1.0 | 350 | 0.001794 | ** |
|  |  |  |  |  |  |  |  |  |  | 500 | 0.00047 | *** |
|  |  |  |  |  |  |  |  | 7.1 | 0.5 | 350 | 0.000899 | *** |
|  |  |  |  |  |  |  |  |  |  | 500 | 3.50E-05 | **** |
|  |  |  |  |  |  |  |  |  | 1.0 | 350 | 7.85E-06 | **** |
|  |  |  |  |  |  |  |  |  |  | 500 | 7.21E-05 | **** |
|  |  |  |  |  |  | 5% | | 6.8 | 0.5 | 350 | 1.22E-06 | **** |
|  |  |  |  |  |  |  |  |  |  | 500 | 5.94E-06 | **** |
|  |  |  |  |  |  |  |  |  | 1.0 | 350 | 0.002037 | ** |
|  |  |  |  |  |  |  |  |  |  | 500 | 7.11E-07 | **** |
|  |  |  |  |  |  |  |  | 7.1 | 0.5 | 350 | 8.68E-06 | **** |
|  |  |  |  |  |  |  |  |  |  | 500 | 0.000204 | *** |
| # | 5% | 6.5 | 1.0 | 500 | | 5% | | 7.1 | 1.0 | 350 | 2.57E-07 | **** |
|  |  |  |  |  |  |  |  |  |  | 500 | 0.000734 | *** |

Table S2. Significant p-values of Tukey’s post-hoc comparison of DNA of goat NP cells encapsulated in alginate beads subjected to various microenvironmental conditions

| Comparison denoted by | Microenvironmental group 1 | | | | compared to: | | Microenvironmental group 2 | | | |  |  |
| --- | --- | --- | --- | --- | --- | --- | --- | --- | --- | --- | --- | --- |
|  | Oxygen (%) | pH | Glucose (mM) | Osmolarity (mOsm) | | Oxygen (%) | | pH | Glucose (mM) | Osmolarity (mOsm) | p-value | Significance |
| & | 5% | 7.1 | 0.5 | 350 | | 2% | | 6.5 | 1.0 | 500 | 0.048496 | * |
|  |  |  |  |  |  | 5% | | 6.5 | 1.0 | 500 | 0.046843 | * |
| % | 2% | 7.1 | 1.0 | 350 | | 2% | | 6.5 | 1.0 | 500 | 0.037103 | * |
|  |  |  |  |  |  | 5% | | 6.5 | 1.0 | 500 | 0.035804 | * |
| $ | 5% | 7.1 | 1.0 | 350 | | 2% | | 6.5 | 1.0 | 500 | 0.04098 | * |
|  |  |  |  |  |  | 5% | | 6.5 | 1.0 | 500 | 0.039559 | * |

Table S3. Significant p-values of Tukey’s post-hoc comparison of Retained GAG/DNADNA of goat NP cells encapsulated in alginate beads subjected to various microenvironmental conditions

| Comparison denoted by | Microenvironmental group 1 | | | | compared to: | | Microenvironmental group 2 | | | |  |  |
| --- | --- | --- | --- | --- | --- | --- | --- | --- | --- | --- | --- | --- |
|  | Oxygen (%) | pH | Glucose (mM) | Osmolarity (mOsm) | | Oxygen (%) | | pH | Glucose (mM) | Osmolarity (mOsm) | p-value | Significance |
| € | 2% | 6.8 | 1.0 | 500 | | 2% | | 6.5 | 0.5 | 350 | 0.03057 | * |
|  |  |  |  |  |  |  |  |  | 1.0 | 350 | 0.044134 | * |
|  |  |  |  |  |  | 5% | | 6.5 | 0.5 | 350 | 0.041176 | * |
| @ | 2% | 7.1 | 1.0 | 500 | | 2% | | 6.5 | 0.5 | 350 | 0.009399 | ** |
|  |  |  |  |  |  |  |  |  |  | 500 | 0.016277 | * |
|  |  |  |  |  |  |  |  |  | 1.0 | 350 | 0.014028 | * |
|  |  |  |  |  |  |  |  |  |  | 500 | 0.022954 | * |
|  |  |  |  |  |  | 5% | | 6.5 | 0.5 | 350 | 0.013002 | * |
|  |  |  |  |  |  |  |  |  |  | 500 | 0.017692 | * |
|  |  |  |  |  |  |  |  |  | 1.0 | 350 | 0.034801 | * |

Table S4. Significant p-values of Tukey’s post-hoc comparison of Oxygen Consumption Rates of goat NP cells encapsulated in alginate beads subjected to various microenvironmental conditions

| Comparison denoted by | Microenvironmental group 1 | | | | compared to: | | Microenvironmental group 2 | | | |  |  |
| --- | --- | --- | --- | --- | --- | --- | --- | --- | --- | --- | --- | --- |
|  | Oxygen (%) | pH | Glucose (mM) | Osmolarity (mOsm) | | Oxygen (%) | | pH | Glucose (mM) | Osmolarity (mOsm) | p-value | Significance |
| + | 5% | 6.5 | 0.5 | 350 | | 2% | | 6.8 | 0.5 | 350 | 0.002481 | ** |
|  |  |  |  |  |  |  |  |  |  | 500 | 0.008642 | ** |
|  |  |  |  |  |  |  |  |  | 1.0 | 350 | 0.009512 | ** |
|  |  |  |  |  |  |  |  |  |  | 500 | 0.002729 | ** |
|  |  |  |  |  |  |  |  | 7.1 | 0.5 | 350 | 0.005885 | ** |
|  |  |  |  |  |  |  |  |  |  | 500 | 0.002943 | ** |
|  |  |  |  |  |  |  |  |  | 1.0 | 350 | 0.010549 | * |
|  |  |  |  |  |  |  |  |  |  | 500 | 0.019765 | * |
|  |  |  |  |  |  | 5% | | 6.8 | 1.0 | 350 | 0.022443 | * |
|  |  |  |  |  |  |  |  |  |  | 500 | 0.004063 | ** |
|  |  |  |  |  |  |  |  | 7.1 | 0.5 | 350 | 0.041428 | * |
|  |  |  |  |  |  |  |  |  | 1.0 | 500 | 0.035328 | * |
| + | 5% | 6.5 | 0.5 | 500 | | 2% | | 6.8 | 0.5 | 350 | 0.002088 | ** |
|  |  |  |  |  |  |  |  |  |  | 500 | 0.007337 | ** |
|  |  |  |  |  |  |  |  |  | 1.0 | 350 | 0.008081 | ** |
|  |  |  |  |  |  |  |  |  |  | 500 | 0.002298 | ** |
|  |  |  |  |  |  |  |  | 7.1 | 0.5 | 350 | 0.004981 | ** |
|  |  |  |  |  |  |  |  |  |  | 500 | 0.00248 | ** |
|  |  |  |  |  |  |  |  |  | 1.0 | 350 | 0.008971 | ** |
|  |  |  |  |  |  |  |  |  |  | 500 | 0.016913 | * |
|  |  |  |  |  |  | 5% | | 6.8 | 1.0 | 350 | 0.019231 | * |
|  |  |  |  |  |  |  |  |  |  | 500 | 0.00343 | ** |
|  |  |  |  |  |  |  |  | 7.1 | 0.5 | 350 | 0.03578 | * |
|  |  |  |  |  |  |  |  |  | 1.0 | 500 | 0.030444 | * |
| £ | 5% | 6.5 | 1.0 | 500 | | 2% | | 6.5 | 0.5 | 500 | 0.001621 | ** |
|  |  |  |  |  |  |  |  |  | 1.0 | 350 | 0.003978 | ** |
|  |  |  |  |  |  |  |  |  |  | 500 | 0.017679 | * |
|  |  |  |  |  |  |  |  | 6.8 | 0.5 | 350 | 1.35E-05 | **** |
|  |  |  |  |  |  |  |  |  |  | 500 | 5.36E-05 | **** |
|  |  |  |  |  |  |  |  |  | 1.0 | 350 | 5.98E-05 | **** |
|  |  |  |  |  |  | 2% | | 6.8 | 1.0 | 500 | 1.49E-05 | **** |
|  |  |  |  |  |  |  |  | 7.1 | 0.5 | 350 | 3.48E-05 | **** |
|  |  |  |  |  |  |  |  |  | 0.5 | 500 | 1.62E-05 | **** |
|  |  |  |  |  |  |  |  |  | 1.0 | 350 | 6.73E-05 | **** |
|  |  |  |  |  |  |  |  |  |  | 500 | 0.00014 | *** |
|  |  |  |  |  |  | 5% | | 6.8 | 0.5 | 350 | 1.35E-05 | *** |
|  |  |  |  |  |  |  |  |  | 1.0 | 350 | 0.000162 | *** |
|  |  |  |  |  |  |  |  |  |  | 500 | 2.31E-05 | **** |
| £ | 5% | 6.5 | 1.0 | 500 | | 5% | | 7.1 | 0.5 | 350 | 0.000342 | *** |
|  |  |  |  |  |  |  |  |  |  | 500 | 0.000711 | *** |
|  |  |  |  |  |  |  |  |  | 1.0 | 350 | 0.001528 | ** |
|  |  |  |  |  |  |  |  |  |  | 500 | 0.000281 | *** |
| ? | 5% | 6.5 | 1.0 | 350 | | 2% | | 6.5 | 0.5 | 350 | 0.013319 | * |
|  |  |  |  |  |  |  |  |  |  | 500 | 0.00032 | *** |
|  |  |  |  |  |  |  |  |  | 1.0 | 350 | 0.000816 | *** |
|  |  |  |  |  |  |  |  |  |  | 500 | 0.003992 | ** |
|  |  |  |  |  |  |  |  | 6.8 | 0.5 | 350 | 2.45E-06 | **** |
|  |  |  |  |  |  |  |  |  |  | 500 | 9.83E-06 | **** |
|  |  |  |  |  |  |  |  |  | 1.0 | 350 | 1.10E-05 | **** |
|  |  |  |  |  |  |  |  |  |  | 500 | 2.71E-06 | **** |
|  |  |  |  |  |  |  |  | 7.1 | 0.5 | 350 | 6.35E-06 | **** |
|  |  |  |  |  |  |  |  |  |  | 500 | 2.95E-06 | **** |
|  |  |  |  |  |  |  |  |  | 1.0 | 350 | 1.24E-05 | **** |
|  |  |  |  |  |  |  |  |  |  | 500 | 2.59E-05 | **** |
|  |  |  |  |  |  | 5% | | 6.8 | 0.5 | 350 | 0.000155 | *** |
|  |  |  |  |  |  |  |  |  |  | 500 | 0.012817 | * |
|  |  |  |  |  |  |  |  |  | 1.0 | 350 | 3.02E-05 | **** |
|  |  |  |  |  |  |  |  |  |  | 500 | 4.20E-06 | **** |
|  |  |  |  |  |  |  |  | 7.1 | 0.5 | 350 | 6.44E-05 | **** |
|  |  |  |  |  |  |  |  |  |  | 500 | 0.000137 | *** |
|  |  |  |  |  |  |  |  |  | 1.0 | 350 | 0.000301 | *** |
|  |  |  |  |  |  |  |  |  |  | 500 | 5.27E-05 | **** |

Table S5. Significant p-values of Tukey’s post-hoc comparison of Lactate Production Rates of goat NP cells encapsulated in alginate beads subjected to various microenvironmental conditions

| Comparison denoted by | Microenvironmental group 1 | | | | compared to: | | Microenvironmental group 2 | | | |  |  |
| --- | --- | --- | --- | --- | --- | --- | --- | --- | --- | --- | --- | --- |
|  | Oxygen (%) | pH | Glucose (mM) | Osmolarity (mOsm) | | Oxygen (%) | | pH | Glucose (mM) | Osmolarity (mOsm) | p-value | Significance |
| ? | 5% | 6.5 | 1.0 | 350 | | 2% | | 6.5 | 0.5 | 350 | 0.021728 | * |
|  |  |  |  |  |  |  |  |  |  | 500 | 0.018518 | * |
|  |  |  |  |  |  |  |  |  | 1.0 | 500 | 0.011607 | * |
|  |  |  |  |  |  |  |  | 6.8 | 0.5 | 350 | 0.02314 | * |
|  |  |  |  |  |  |  |  |  | 1.0 | 350 | 0.004493 | ** |
|  |  |  |  |  |  |  |  | 7.1 | 0.5 | 350 | 0.012985 | * |
|  |  |  |  |  |  |  |  |  |  | 500 | 0.003734 | ** |
|  |  |  |  |  |  |  |  |  | 1.0 | 350 | 0.008194 | ** |
|  |  |  |  |  |  |  |  |  |  | 500 | 0.010212 | * |
| ε | 5% | 6.8 | 1.0 | 350 | | 2% | | 6.5 | 0.5 | 350 | 0.002467 | ** |
|  |  |  |  |  |  |  |  |  |  | 500 | 0.002065 | ** |
|  |  |  |  |  |  |  |  |  | 1.0 | 350 | 0.035549 | * |
|  |  |  |  |  |  |  |  |  |  | 500 | 0.001234 | ** |
|  |  |  |  |  |  |  |  | 6.8 | 0.5 | 350 | 0.002648 | ** |
|  |  |  |  |  |  |  |  |  | 1.0 | 350 | 0.000442 | *** |
|  |  |  |  |  |  |  |  |  |  | 500 | 0.015621 | * |
|  |  |  |  |  |  |  |  | 7.1 | 0.5 | 350 | 0.001395 | ** |
|  |  |  |  |  |  |  |  |  |  | 500 | 0.000363 | *** |
|  |  |  |  |  |  |  |  |  | 1.0 | 350 | 0.000844 | *** |
|  |  |  |  |  |  |  |  |  |  | 500 | 0.001073 | ** |
|  |  |  |  |  |  | 5% | | 6.5 | 0.5 | 350 | 0.009269 | ** |
| & | 5% | 7.1 | 0.5 | 350 | | 2% | | 6.8 | 1.0 | 350 | 0.034454 | * |
|  |  |  |  |  |  |  |  | 7.1 | 0.5 | 500 | 0.02925 | * |
| ψ | 5% | 6.8 | 0.5 | 350 | | 2% | | 6.5 | 0.5 | 350 | 0.029755 | * |
|  |  |  |  |  |  |  |  |  |  | 500 | 0.025459 | * |
|  |  |  |  |  |  |  |  |  | 1.0 | 500 | 0.016122 | * |
|  |  |  |  |  |  |  |  | 6.8 | 0.5 | 350 | 0.031639 | * |
|  |  |  |  |  |  |  |  |  | 1.0 | 350 | 0.006346 | ** |
|  |  |  |  |  |  |  |  | 7.1 | 0.5 | 350 | 0.017994 | * |
|  |  |  |  |  |  |  |  |  |  | 500 | 0.005287 | ** |
|  |  |  |  |  |  |  |  |  | 1.0 | 350 | 0.011458 | * |
|  |  |  |  |  |  |  |  |  |  | 500 | 0.01422 | * |
| $ | 5% | 7.1 | 1.0 | 350 | | 2% | | 6.5 | 0.5 | 350 | 0.000318 | *** |
|  |  |  |  |  |  |  |  |  |  | 500 | 0.000264 | *** |
|  |  |  |  |  |  |  |  |  | 1.0 | 350 | 0.005713 | ** |
|  |  |  |  |  |  |  |  |  |  | 500 | 0.000154 | *** |
|  |  |  |  |  |  |  |  | 6.8 | 0.5 | 350 | 0.000343 | *** |
|  |  |  |  |  |  |  |  |  | 0.5 | 500 | 0.015552 | * |
|  |  |  |  |  |  |  |  |  | 1.0 | 350 | 5.33E-05 | **** |
|  |  |  |  |  |  |  |  |  |  | 500 | 0.002294 | ** |
|  |  |  |  |  |  |  |  | 7.1 | 0.5 | 350 | 0.000175 | *** |
|  |  |  |  |  |  |  |  |  |  | 500 | 4.35E-05 | **** |
|  |  |  |  |  |  |  |  |  | 1.0 | 350 | 0.000104 | *** |
|  |  |  |  |  |  |  |  |  |  | 500 | 0.000133 | *** |
|  |  |  |  |  |  | 5% | | 6.5 | 0.5 | 350 | 0.001301 | ** |
|  |  |  |  |  |  |  |  |  |  | 500 | 0.011428 | * |
| £ | 5% | 6.5 | 1.0 | 500 | | 2% | | 6.5 | 1.0 | 500 | 0.042912 | * |
|  |  |  |  |  |  |  |  | 6.8 | 1.0 | 350 | 0.017981 | * |
|  |  |  |  |  |  |  |  | 7.1 | 0.5 | 350 | 0.04748 | * |
| £ | 5% | 6.5 | 1.0 | 500 | | 2% | | 7.1 | 0.5 | 500 | 0.015136 | * |
|  |  |  |  |  |  |  |  |  | 1.0 | 350 | 0.031271 | * |
|  |  |  |  |  |  |  |  |  |  | 500 | 0.038211 | * |
| σ | 5% | 7.1 | 0.5 | 500 | | 2% | | 6.8 | 1.0 | 350 | 0.04731 | * |
|  |  |  |  |  |  |  |  | 7.1 | 0.5 | 500 | 0.040362 | * |
